# Integrative DNA methylation and gene expression analysis in high-grade soft tissue sarcomas

**DOI:** 10.1186/gb-2013-14-12-r137

**Published:** 2013-12-17

**Authors:** Marcus Renner, Thomas Wolf, Hannah Meyer, Wolfgang Hartmann, Roland Penzel, Alexis Ulrich, Burkhard Lehner, Volker Hovestadt, Esteban Czwan, Gerlinde Egerer, Thomas Schmitt, Ingo Alldinger, Eva Kristin Renker, Volker Ehemann, Roland Eils, Eva Wardelmann, Reinhard Büttner, Peter Lichter, Benedikt Brors, Peter Schirmacher, Gunhild Mechtersheimer

**Affiliations:** 1 Department of General Pathology, Institute of Pathology, University Hospital Heidelberg, Im Neuenheimer Feld 224, 69120 Heidelberg, Germany; 2 Theoretical Bioinformatics, German Cancer Research Center (DKFZ), 69120 Heidelberg, Germany; 3 Institute of Pathology, University Hospital of Cologne, 50937 Cologne, Germany; 4 Department of General, Visceral and Transplantation Surgery, University Hospital Heidelberg, 69120 Heidelberg, Germany; 5 Division of Orthopedic Oncology, Department of Orthopedics, Trauma Surgery and Paraplegiology, University Hospital Heidelberg, 69118 Heidelberg, Germany; 6 Division of Molecular Genetics, German Cancer Research Center (DKFZ), 69120 Heidelberg, Germany; 7 Department of Hematology, Oncology, and Rheumatology, University Hospital Heidelberg, 69120 Heidelberg, Germany; 8 Department of Bioinformatics and Functional Genomics, Institute of Pharmacy and Molecular Biotechnology, Bioquant, University of Heidelberg, 69120 Heidelberg, Germany

## Abstract

**Background:**

High-grade soft tissue sarcomas are a heterogeneous, complex group of aggressive malignant tumors showing mesenchymal differentiation. Recently, soft tissue sarcomas have increasingly been classified on the basis of underlying genetic alterations; however, the role of aberrant DNA methylation in these tumors is not well understood and, consequently, the usefulness of methylation-based classification is unclear.

**Results:**

We used the Infinium HumanMethylation27 platform to profile DNA methylation in 80 primary, untreated high-grade soft tissue sarcomas, representing eight relevant subtypes, two non-neoplastic fat samples and 14 representative sarcoma cell lines. The primary samples were partitioned into seven stable clusters. A classification algorithm identified 216 CpG sites, mapping to 246 genes, showing different degrees of DNA methylation between these seven groups. The differences between the clusters were best represented by a set of eight CpG sites located in the genes *SPEG*, *NNAT*, *FBLN2*, *PYROXD2*, *ZNF217*, *COL14A1*, *DMRT2* and *CDKN2A*. By integrating DNA methylation and mRNA expression data, we identified 27 genes showing negative and three genes showing positive correlation. Compared with non-neoplastic fat, *NNAT* showed DNA hypomethylation and inverse gene expression in myxoid liposarcomas, and DNA hypermethylation and inverse gene expression in dedifferentiated and pleomorphic liposarcomas. Recovery of *NNAT* in a hypermethylated myxoid liposarcoma cell line decreased cell migration and viability.

**Conclusions:**

Our analysis represents the first comprehensive integration of DNA methylation and transcriptional data in primary high-grade soft tissue sarcomas. We propose novel biomarkers and genes relevant for pathogenesis, including *NNAT* as a potential tumor suppressor in myxoid liposarcomas.

## Background

The role of aberrant DNA methylation in the development of human malignancies is well established and has been shown to contribute to the pathogenesis of cancer [1,2]. There is strong evidence suggesting a relation between the presence of CpG island methylation and the level of target gene expression [3]. In particular, the increased methylation of DNA in 5’ upstream regulatory sites shows negative correlation with gene expression of some tumor-suppressor genes, suggesting that alterations of DNA methylation can be exploited for functional characterization and diagnosis of cancer [4,5]. In contrast, several instances have been observed where the correlation between methylation status and gene expression does not follow these established hypotheses [6]. High levels of gene body methylation have been positively correlated with an increase in gene expression [7]. Hypermethylation has been assumed as a silencing mechanism for tumor suppressor genes, developmental programs and imprinting [8,9], and as crucial for maintaining cell differentiation and fate [10,11]. Human cell lines, which are commonly used for *in vitro* studies of primary tumors, show distinctly higher levels of CpG island hypermethylation than their corresponding primary tumors [12,13].

Soft tissue sarcomas (STSs) are a group of highly aggressive, histologically and genetically heterogeneous malignant tumors of mesenchymal origin. They occur almost anywhere in the human body and account for approximately 1% of all adult malignancies. Sarcomas can be classified histologically according to the soft tissue cell of origin. Myxoid/round cell liposarcomas (MLSs), dedifferentiated liposarcomas (DDLSs) and pleomorphic liposarcomas (PLSs) are adipocytic tumors. Leiomyosarcomas (LMSs) are smooth muscle tumors, and malignant peripheral nerve sheath tumors (MPNSTs) arise from the Schwann cells of peripheral nerves. Undifferentiated high-grade pleomorphic sarcomas (UPSs) belong to the heterogeneous group of fibrohistiocytic tumors. It is proposed that myxofibrosarcomas (MFSs) are myxoid variants of UPS. Since the cellular origin of synovial sarcomas (SSs) is still unknown, these tumor belongs to sarcomas of uncertain differentiation.

Another classification is based on genetic alterations. According to this, sarcomas can be classified into two main groups: (a) sarcomas with specific genetic alterations on a background of relatively few chromosomal changes and (b) sarcomas with no specific genetic alterations on a complex background of numerous chromosomal changes. One third of sarcomas belongs to the first group, characterized by specific and recurrent chromosomal translocations [14]. For example, SSs and MLSs are characterized by subtype-specific translocations, gastrointestinal stromal tumors (GISTs) carry *KIT* gene mutations, well-differentiated liposarcomas (WDLSs) and DDLSs show amplifications of the *MDM2* gene, and extrarenal rhabdoid tumors have a high incidence of homozygous deletions of the *SMARCB1* gene [15]. Examples of sarcomas with complex chromosomal changes are PLS, UPS, MFS, LMS and MPNST.

Only a few diagnostic and prognostic markers exist, and the cellular origin of several sarcoma subtypes is unknown. Therefore, the accurate diagnosis and the prediction of the clinical behavior of many of these tumors remain a challenge [16]. High-grade sarcomas show high rates of local recurrence, frequent metastasis and poor prognosis [17]. The main treatment is surgery with complete and wide excision. Despite improvements in local tumor control by surgery, radiotherapy and chemotherapy, distant metastasis and high tumor-related lethality remain problems of current treatment strategies [18,19]. Hence, new strategies for the treatment of patients with soft tissue sarcomas are urgently needed. Microarray-based CGH and expression profiling of mRNAs and miRNAs have identified genomic alterations, candidate genes and miRNAs, which can be used to discriminate sarcoma subtypes and to determine disease progression and they are potential therapeutic targets [20-25].

While previous studies have profiled DNA methylation in soft tissue sarcomas [26-31], they have either been limited to specific sarcoma subtypes or genes with corresponding CpG islands or sites. Genome-wide DNA methylation studies suggest there are distinct DNA methylation patterns in pediatric embryonal and alveolar rhabdomyosarcomas [26] and they have revealed genes that are potential targets of epigenetic inactivation in Ewing’s sarcoma [32]. Bisulfite sequencing-based methylome analysis of a primary and recurrent dedifferentiated liposarcoma identified alterations in differentiation pathway genes, including *CEBPA*, a transcriptional regulator of adipocyte differentiation [30]. While epigenetic abnormalities have been extensively characterized in STSs, their influence on mRNA expression in a large cohort of primary, high-grade sarcoma samples has not been described in a genome-wide study so far. This limits the ability to identify a subtype-specific DNA methylation signature for sarcoma classification and a set of candidate methylation-responsive genes linked to changes in gene expression. To address these issues, we performed genome-wide DNA methylation profiling using the Illumina Infinium HumanMethylation27 platform for a collection of 80 primary and untreated high-grade STS samples representing eight different sarcoma subtypes, two non-neoplastic fat samples and 14 corresponding and representative sarcoma cell lines. We integrated our methylation data with mRNA expression data to identify diagnostically relevant DNA methylation changes between different sarcoma subtypes and functional relevant genes including potential tumor-suppressor candidates. Our results suggest that DNA methylation signatures may aid in the diagnosis and risk stratification of high-grade STSs and help to identify new candidates and targets for therapy.

## Results

### DNA methylation profiles of 80 primary high-grade soft tissue sarcomas

Using the HumanMethylation27 BeadChip platform, we interrogated the DNA methylation status of a collection of 80 primary and untreated high-grade STSs (Additional file 1: Table S1), two non-neoplastic fat tissue samples and 14 sarcoma cell lines (Additional file 1: Table S2). Of the 27,578 probes on the chip, 1,737 showed a clear bimodal hypo-/hypermethylation *M* value distribution [33]. Probes not showing such bimodal hypo-/hypermethylation patterns were excluded from further analysis. The selected set of probes formed the basis for all further analysis steps. This *M*-value-based binarization translates to mean beta values (over all samples including primary tumors, non-neoplastic fat cells and cell lines) of 0.14 (SD 0.11) and 0.64 (SD 0.13) for hypo- and hypermethylation, respectively. Of the selected CpG sites, 174 were located on the X chromosome. All other CpGs on chromosomes X and Y were excluded.

### Unsupervised clustering using observed methylation patterns

**Figure 1:**
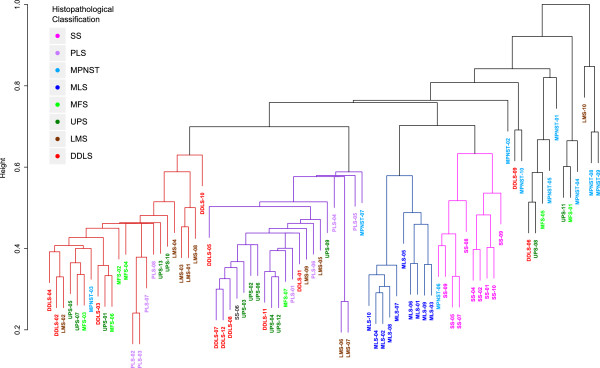
**Unsupervised hierarchical clustering.** The figure shows the unsupervised hierarchical clustering of the DNA methylation data for 80 primary high-grade STS samples representing eight sarcoma subtypes. The two sarcoma subtypes that carry specific translocations (MLS and SS) are tightly separated into subgroups. The other sarcoma subtypes were dispersed into a third or fourth subgroup. DDLS, dedifferentiated liposarcoma; LMS, leiomyosarcoma; MFS, myxofibrosarcoma; MLS, myxoid liposarcoma; MPNST, malignant peripheral nerve sheath tumor; PLS, pleomorphic liposarcoma; SS, synovial sarcoma; UPS, undifferentiated pleomorphic sarcoma.

To get an overview of how well the histopathologic sarcoma subtypes are reflected on the DNA methylation level, we performed unsupervised cluster analysis of the 1,737 probes that showed clear bimodal hypo-/hypermethylation patterns. The hypermethylation binary definition did not make any use of sarcoma subtype information, and is based entirely on the DNA methylation signal. The dendrogram obtained by divisive hierarchical clustering [34] revealed four main sarcoma subgroups (Figure 1). Two subgroups consisted exclusively of the two translocation-associated sarcoma subtypes, MLS and SS. Only one MPNST sample clustered in close proximity to the SS group and had a DNA methylation pattern similar to that of the SS samples. The two remaining subgroups were composed of the six other sarcoma subtypes in a heterogeneous manner. Some sarcoma samples had completely different DNA methylation profiles and did not cluster with the four subgroups. Of interest, seven of the ten MPNST samples belong to this group.

### Supervised classification of histopathological sarcoma subtypes

Since the unsupervised approach did not use feature weighting or non-linear combinations between features, not all subtypes could be clearly separated on the DNA methylation level. Some subtypes may still show similarity only for a subset of DNA methylation sites, while being distinctly heterogeneous over the entire set of probes. To address these points, a supervised random forest (RF) model was trained using the histopathological subtype classification. The classification was assessed using both the random forest out-of-the-bag (OOB) error (Table 1a) and ten repeats of class-stratified tenfold cross validation (Additional file 1: Table S3). According to the OOB error, the classification had an overall accuracy of 70% (ten repeats of class-stratified tenfold cross validation, 73% accuracy) and mostly separated MLS, SS, LMS and DDLS samples (Table 1b). These results suggest that the histopathological groups are reflected on the DNA methylation level, but also indicate that a methylation-based regrouping would show distinct differences from established diagnostics. For instance UPSs, MFSs, and PLSs could not be clearly separated, but the confusion matrix (Table 1b) supports a subgroup mainly composed of PLSs and MFSs and another group including only MFS and UPS samples. The confusion matrix has only a reduced level of information necessary for such a regrouping. Thus we made use of the proximity measure obtained from the RF model.

**Table 1:** Accuracy of the histopathological subtype classification

**a**								
**Class**	**Sarcoma subtype**	**Sensitivity**	**Specificity**	**Positive prediction value**	**Negative prediction value**			
1	DDLS	0.83	0.96	0.77	0.97			
2	LMS	0.80	1.00	1.00	0.97			
3	PLS	0.38	0.94	0.43	0.93			
4	UPS	0.38	0.87	0.36	0.88			
5	MFS	0.29	0.95	0.33	0.93			
6	MLS	1.00	0.99	0.91	1.00			
7	MPNST	0.80	0.97	0.80	0.97			
8	SS	1.00	0.99	0.91	1.00			
**b**								
	**Reference**							
**Prediction**	**DDLS**	**LMS**	**PLS**	**UPS**	**MFS**	**MLS**	**MPNST**	**SS**
DDLS	10	0	1	2	0	0	0	0
LMS	0	8	0	0	0	0	0	0
PLS	0	1	3	3	0	0	0	0
UPS	1	0	4	5	4	0	0	0
MFS	0	0	0	3	2	0	1	0
MLS	0	0	0	0	1	10	0	0
MPNST	1	1	0	0	0	0	8	0
SS	0	0	0	0	0	0	1	10

Overall accuracy: 0.70.

The column “MLS” is hidden between “MFS” and “MPNST”.

DDLS, dedifferentiated liposarcoma; LMS, leiomyosarcoma; MFS, myxofibrosarcoma; MLS, myxoid liposarcoma; MPNST, malignant peripheral nerve sheath tumor; PLS, pleomorphic liposarcoma; SS, synovial sarcoma; UPS, undifferentiated pleomorphic sarcoma.

### Model analysis I: methylation-based regrouping of histopathological sarcoma subtypes

**Figure 2:**
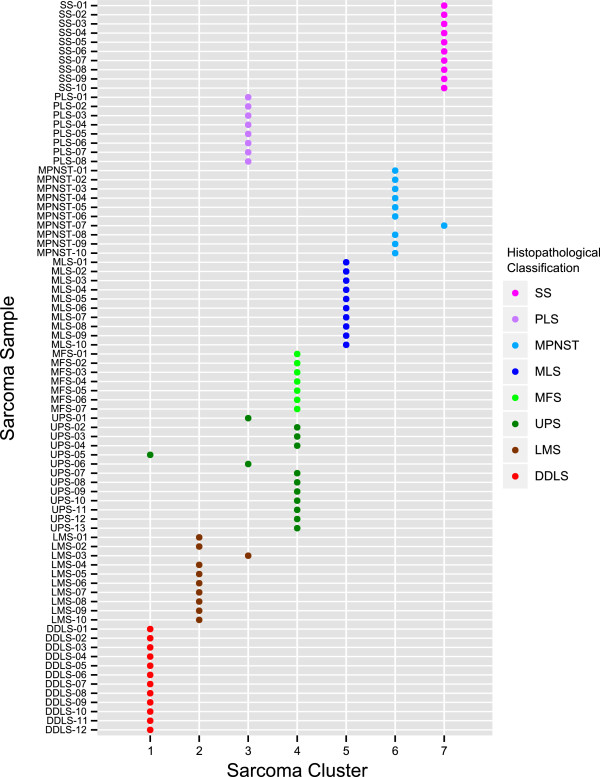
**Identification of seven stable methylation clusters.** Seven stable methylation clusters were identified in the STS collection using a random forest clustering approach that integrated histopathological groupings and DNA methylation patterns. DDLS, dedifferentiated liposarcoma; LMS, leiomyosarcoma; MFS, myxofibrosarcoma; MLS, myxoid liposarcoma; MPNST, malignant peripheral nerve sheath tumor; PLS, pleomorphic liposarcoma; SS, synovial sarcoma; STS, soft tissue sarcoma; UPS, undifferentiated pleomorphic sarcoma.

To get a more in-depth overview than can be obtained from the confusion matrix, we made use of the proximity as returned by the RF model. This proximity can be considered unbiased, as the pairwise proximity was only calculated over single trees for which both samples were not part of the training set. The samples were clustered into eight groups using the partitioning around medoids algorithm (PAM) and the stability of each cluster was assessed by bootstrapping the proximity matrix [35]. Of the eight sarcoma clusters (Additional file 2: Figure S1) two clusters contained mainly MFS and UPS samples and were not considered stable (Additional file 1: Tables S3 and S4). Since it is proposed that myxofibrosarcomas are myxoid variants of UPS, we combined these two sarcoma subtypes (Figure 2). Based on the final seven clusters, most UPS and MFS samples were grouped into one combined cluster (sarcoma cluster 4). SS and MLS samples each formed a distinct cluster without exception. All other subtypes composed sarcoma clusters according to their histopathological classification and only a few samples grouped with other sarcoma clusters. Two UPS samples and one LMS sample were in the PLS cluster, sarcoma cluster 3. A third UPS sample was in the DDLS cluster (sarcoma cluster 1) and one MPNST sample was in the cluster composed of SS samples (sarcoma cluster 7). These samples were histologically re-evaluated. However, none of these samples was reclassified. As this study focuses on DNA methylation profiles instead of just histopathological subtypes, these seven stable sarcoma clusters represented the basis for all further analyses.

### Model analysis II: analysis of cluster-based DNA methylation patterns

To explore the differential DNA methylation patterns that define these clusters [36], a supervised RF model was generated. Of the 1,737 probes that showed clear bimodal hypo-/hypermethylation patterns, 880 were significant (*P* ≤ 0.05) according to a univariate Kruskal–Wallis test adjusted for multiple testing using the Benjamini–Hochberg approach. Of these, 216 CpG probes were selected as informative (95% confidence) using the Boruta method (Additional file 1: Tables S6 and S7). The model (RF classifier) and feature selection (Kruskal–Wallis and Boruta) were assessed using ten times tenfold cross validation. All clustering (RF and PAM) and feature selection steps using class information (Kruskal–Wallis and Boruta) were included in the stratified cross validation (see Additional file 2: Figure S2 for a detailed description of the cross-validation procedure). The overall accuracy was 0.82 and the highest sensitivity and specificity were obtained for LMS and the two translocation-related subtypes, MLS (cluster 5 included all MLS samples) and SS (cluster 7 included all SS samples and one MPNST sample; Table 2, Additional file 1: Table S5). Annotation of this CpG set identified 249 corresponding genes since some CpG sites map to more than one gene (count annotation, Additional file 1: Table S6). These 216 CpG sites served as the basis for all further analysis steps. Of the CpG sites, 74% (*n* = 165) sites were located in CpG islands and two were located on the X chromosome. However, the DNA methylation changes of these two CpG sites were MLS specific and not due to gender (Additional file 2: Figure S3a and b).

**Table 2:** Accuracy of the multivariate classifier

**Sarcoma cluster**	**Sarcoma subtype**	**Sensitivity**	**Specificity**	**Positive prediction value**	**Negative prediction value**
1	DDLS	0.75	0.97	0.83	0.95
2	LMS	1.00	1.00	1.00	1.00
3	PLS	0.72	0.96	0.72	0.96
4	UPS/MFS	0.68	0.88	0.61	0.91
5	MLS	1.00	1.00	1.00	1.00
6	MPNST	0.82	0.99	0.90	0.98
7	SS	0.91	0.99	0.91	0.99

Overall accuracy: 0.82.

DDLS, dedifferentiated liposarcoma; LMS, leiomyosarcoma; MFS, myxofibrosarcoma; MLS, myxoid liposarcoma; MPNST, malignant peripheral nerve sheath tumor; PLS, pleomorphic liposarcoma; SS, synovial sarcoma; UPS, undifferentiated pleomorphic sarcoma.

**Figure 3:**
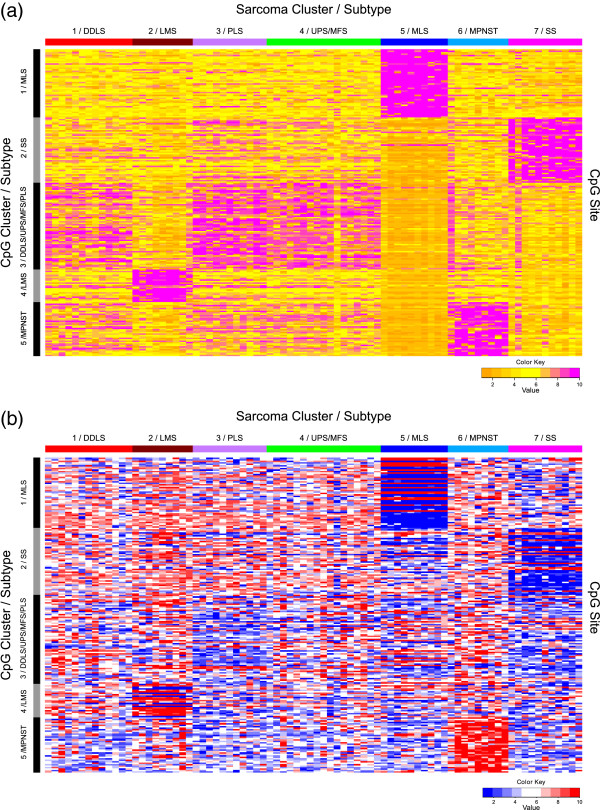
**CpG sites selected by the Boruta method.** Local importance **(a)** and *M* values **(b)** of the 216 CpG sites grouped into deciles. The CpG sites shown were selected by the Boruta method as being differential between the seven sarcoma clusters identified by random forest clustering. The seven sarcoma clusters are given in columns and CpG sites are given in rows. A characteristic set of CpG sites was identified for sarcoma cluster 2 (LMS samples), cluster 5 (MLS samples), cluster 6 (MPNST samples) and cluster 7 (SS samples including one MPNST sample). The sarcoma clusters 1, 3 and 4 (mainly DDLS, UPS, MFS and PLS samples) had different patterns of the same set of CpGs and composed CpG cluster 3. The order of the CpG sites is listed in Additional file 1: Table S6. The color code for DNA methylation level is given at the bottom of each graph. For local importance (a), yellow indicates low and purple indicates high importance. DDLS, dedifferentiated liposarcoma; LMS, leiomyosarcoma; MFS, myxofibrosarcoma; MLS, myxoid liposarcoma; MPNST, malignant peripheral nerve sheath tumor; PLS, pleomorphic liposarcoma; SS, synovial sarcoma; UPS, undifferentiated pleomorphic sarcoma.

**Figure 4:**
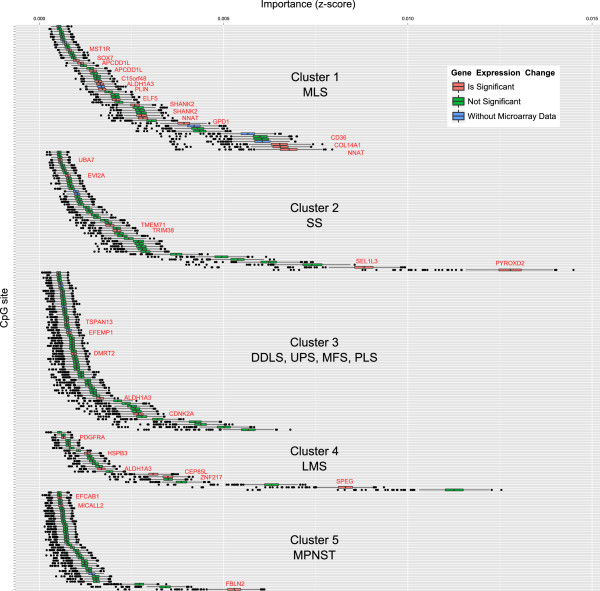
**Boruta plot showing the importance (*x*-axis) of each CpG site for the five CpG clusters.** The 35 CpG sites from the preselected set of 216 CpG sites (*y*-axis) that met the inclusion criteria for a reliable influence on gene expression (Table 3) are labeled with the annotated gene name and are highlighted in red. The order of the CpG sites is listed in Additional file 1: Table S6. CpG sites are marked in green when they did not meet the inclusion criteria. CpG sites without gene expression data are marked in blue. DDLS, dedifferentiated liposarcoma; LMS, leiomyosarcoma; MFS, myxofibrosarcoma; MLS, myxoid liposarcoma; MPNST, malignant peripheral nerve sheath tumor; PLS, pleomorphic liposarcoma; SS, synovial sarcoma; UPS, undifferentiated pleomorphic sarcoma.

In addition to the global variable importance, RF also calculates the local variable importance. This gives an estimate of the importance of a variable in the classification of a single sample. Thus, an importance value is estimated for each variable/sample combination. Based on these values, we identified five CpG subgroups using PAM clustering, showing similar local importance patterns over all sarcoma samples. The identified CpG clusters were clearly associated with the seven sarcoma clusters (Figure 3a, b). These CpG subgroups had the highest importance when classifying members of the respective sarcoma subgroups correctly (Additional file 2: Figure S3c). A characteristic set of CpG sites was identified for each of the four sarcoma subtypes MLS, LMS, SS and MPNST. DDLS, UPS, MFS and PLS had different patterns of the same set of CpG sites (Figure 3a, b). CpG cluster 1 (MLS samples) contained 48 CpG sites, CpG cluster 2 (mainly SS samples) 46 CpG sites, CpG cluster 4 (LMS samples) 23 CpG sites and 38 CpG sites were characteristic for CpG cluster 5 (MPNST samples). CpG cluster 3 incorporated 61 CpG sites mainly associated with DDLS, UPS, MFS and PLS subtypes. All 216 CpG sites were given a short name that was composed of the CpG cluster information and a consecutive number reflecting the importance for each CpG cluster (Additional file 1: Table S6). The order and distribution of the feature importance, as obtained from the Boruta method, is shown in Figure 4.

### Identification of functionally relevant DNA methylation changes in sarcomas

To investigate the correlation between DNA methylation status and gene expression, we carried out expression profiling of the same collection of primary high-grade STS samples and integrated the two data sets. Finally, both data sets were available from 79 of the 80 sarcoma samples. Of the preselected 216 CpG sites, we identified a significant (*P* ≤ 0.05, Kendall correlation) negative correlation for 48 CpG sites and significant positive correlation for 13 CpG sites. This suggests that aberrant DNA methylation might have functional consequences in approximately 25% of the genes showing differential DNA methylation for the seven sarcoma clusters. To identify stable gene expression changes due to DNA methylation status, several constraints had to be met. These included a 1.5-fold gene expression change together with a significant *P* < 0.05 (Wilcoxon rank sum test, adjusted for multiple testing using the Benjamini–Hochberg approach over all comparisons made for the preselected 216 CpG sites) between hypo- and hypermethylated conditions, as well as a significant correlation between DNA methylation level and gene expression (*P* ≤ 0.05, Kendall correlation, adjusted for multiple testing using the Benjamini–Hochberg approach over all correlations calculated for the 216 preselected CpG sites). The direction of the correlation had to be the same as for the detected DNA methylation fold change. Of the CpG sites, 35 met these criteria; four sites showed positive and 31 sites showed negative correlation (Table 3, Additional file 1: Table S8). These CpG sites could be annotated to 30 corresponding genes since some CpG sites were annotated to the same gene.

**Table 3:** Characteristics of the selected CpG sites

**Short name**	**CpG number**	**Gene symbol**	**1/FC**	**1/FC**	**Correlation**	**CpG region**	**Hypomethylated**	**Hypermethylated**	**Absolute d**	**Kruskal–Wallis**
			**(Gene expression)**	**(Methylation)**			**(β value)**	**(β value)**	**(β value)**	***P *** **value**
**Negative correlation**										
4–09	cg19510698	*ALDH1A3*	6.3	6.9	-0.49	GB	0.339	0.748	0.41	3.5 × 10^–5^
3–13	cg27652350	*ALDH1A3*	6.0	31.4	-0.44^a^	GB	0.181	0.830	0.65	1.5 × 10^–5^
1–26	cg21359747	*ALDH1A3*	5.7	28.8	-0.38	P	0.121	0.751	0.63	2.5 × 10^–5^
2–37	cg23352695	*EVI2A*	1.8	8.4	-0.47^a^	P	0.235	0.694	0.46	6.5 × 10^–5^
3–30	cg00250430	*DMRT2*	2.7	14.5	-0.41^b^	P	0.138	0.652	0.51	1.2 × 10^–4^
2–18	cg20955688	*TMEM71*	1.6	11.6	-0.41	P	0.090	0.490	0.40	5.1 × 10^–5^
1–39	cg08687163	*MST1R*	1.6	19.0	-0.39^b^	P	0.566	0.951	0.39	1.2 × 10^–4^
2–43	cg09874127	*UBA7*	4.5	6.8	-0.37	P	0.094	0.406	0.31	1.6 × 10^–3^
1–34	cg23418591	*APCDD1L*	5.9	36.6	-0.33	P	0.097	0.739	0.64	5.8 × 10^–5^
1–31	cg14546153	*APCDD1L*	5.2	22.2	-0.33	P	0.057	0.549	0.49	1.1 × 10^–5^
5–01	cg00201234	*FBLN2*	3.4	31.9	-0.32	P	0.075	0.662	0.59	1.1 × 10^–4^
4–21	cg22736323	*PDGFRA*	6.3	11.7	-0.31	P	0.182	0.679	0.50	4.8 × 10^–2^
1–24	cg01035422	*PLIN*	4.9	5.0	-0.31	P	0.167	0.544	0.38	1.4 × 10^–3^
1–01	cg22298088	*NNAT*	24.7	9.3	-0.30^b^	P	0.492	0.876	0.38	1.1 × 10^–5^
1–13	cg12862537	*NNAT*	41.4	16.6	-0.27	P	0.548	0.940	0.39	2.0 × 10^–4^
2–16	cg22502502	*TRIM38*	1.7	7.2	-0.30	P	0.120	0.468	0.35	1.1 × 10^–2^
5–36	cg22836229	*EFCAB1*	1.6	16.1	-0.30	P	0.167	0.708	0.54	3.0 × 10^–3^
4–15	cg11391732	*HSPB3*	2.4	9.4	-0.30	P	0.361	0.795	0.43	6.7 × 10^–3^
1–03	cg18508525	*CD36*	13.5	17.4	-0.30	P	0.246	0.811	0.56	9.7 × 10^–5^
3–38	cg20786074	*EFEMP1*	1.9	11.0	-0.28^a^	P	0.167	0.658	0.49	6.0 × 10^–5^
2–02	cg10150813	*SEL1L3*	1.5	14.1	-0.27	P	0.232	0.771	0.54	9.6 × 10^–5^
1–27	cg08278554	*C15orf48*	1.7	10.1	-0.27	P	0.183	0.640	0.46	1.3 × 10^–5^
2–01	cg08397758	*PYROXD2*	1.6	17.4	-0.26	P	0.159	0.709	0.55	3.2 × 10^–4^
4–06	cg00476577	*ZNF217*	1.6	9.7	-0.26	P	0.094	0.486	0.39	1.9 × 10^–4^
1–02	cg16907566	*COL14A1*	1.7	13.8	-0.26	P	0.120	0.606	0.49	9.6 × 10^–5^
1–20	cg01473816	*ELF5*	2.3	4.2	-0.25	P	0.461	0.758	0.30	6.3 × 10^–4^
1–11	cg25181284	*GPD1*	3.6	9.9	-0.25	P	0.524	0.910	0.39	1.5 × 10^–4^
4–07	cg26205432	*PLN*	3.4	3.8	-0.24	P	0.302	0.581	0.28	3.1 × 10^–3^
3–42	cg12567315	*TSPAN13*	1.5	19.8	-0.23	P	0.053	0.494	0.44	1.6 × 10^–2^
4–02	cg10062065	*SPEG*	2.4	15.2	-0.20	P	0.364	0.870	0.51	7.3 × 10^–4^
***Positive correlation***										
3–07	cg10895543	*CDKN2A*	0.3	17.9	0.58	GB	0.188	0.758	0.57	2.8 × 10^–6^
1–35	cg24690731	*SOX7*	0.6	12.1	0.30	P	0.217	0.735	0.52	4.3 × 10^–4^
1–14	cg04396791	*SHANK2*	0.4	52.7	0.25	P	0.079	0.759	0.68	8.9 × 10^–4^
1–18	cg10362475	*SHANK2*	0.5	18.1	0.25	GB	0.221	0.792	0.57	8.0 × 10^–4^
5–33	cg01820777	*MICALL2*	0.6	5.3	0.20	P	0.089	0.345	0.26	7.8 × 10^–3^

^a^Significant correlation of gene expression within the range of DNA hypermethylation.

^b^Significant correlation of gene expression within the range of DNA hypomethylation.

GB, gene body; P, promoter.

1/FC is defined as the inverse value of the fold change.

**Figure 5:**
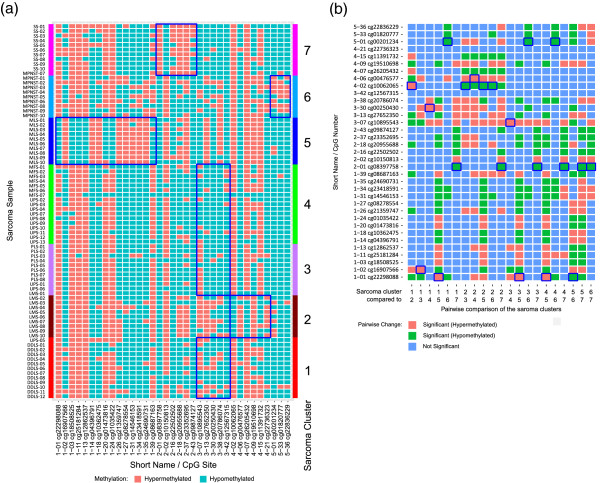
**Binarized DNA methylation status and pairwise comparison of the CpG sites. (a)** Binarized DNA methylation status of the 35 CpG sites showing a stable influence on gene expression over the samples of the primary sarcoma collection. The sarcoma cluster assignment from the initial random forest clustering (Figure 2) is indicated by the colored bars. The CpG short names contain the CpG cluster number together with the importance. Cluster-specific CpG sites are bordered in blue. Hypermethylation and hypomethylation are shown in red and blue, respectively. **(b)** Pairwise comparison of the 35 preselected CpG sites for the seven sarcoma clusters. One representative CpG site was selected for each comparison (bordered in blue). Significant hyper- and hypomethylation of CpG sites for the two sarcoma clusters are marked in red and green, respectively (Wilcoxon rank sum test). Unchanged CpG sites are indicated in blue. DDLS, dedifferentiated liposarcoma; LMS, leiomyosarcoma; MFS, myxofibrosarcoma; MLS, myxoid liposarcoma; MPNST, malignant peripheral nerve sheath tumor; PLS, pleomorphic liposarcoma; SS, synovial sarcoma; UPS, undifferentiated pleomorphic sarcoma.

To obtain an overview of the cluster-wise importance of the 35 functionally relevant CpG sites, they were labeled with the gene name and highlighted in red in a Boruta plot, which shows the importance of all preselected 216 CpG sites in the differentiation of the five CpG clusters (Figure 4). Of interest, there was a reliable link between the genes with highest importance for sarcoma cluster 1 (MLS samples), cluster 2 (mainly SS samples) and cluster 5 (MPNST samples) and gene expression according to the applied criteria. For the three genes with the highest importance in the MLS cluster, that is, *NNAT*, *COL14A1* and *CD36*, there was a correlation between DNA methylation and gene expression. The DNA methylation status of the 35 CpG sites for each sample in the sarcoma collection is detailed in Figure 5a and Additional file 2: Figure S4. Furthermore, we analyzed the correlation between probe-wise DNA methylation and gene expression separately for the samples showing hypo- or hypermethylation according to the binarization (Table 3 and Additional file 1: Table S8). Three CpG sites (*ALDH1A3*, *EVI2A* and *EFEMP1*) had a significant correlation for the hypermethylated samples and three CpG sites (*DMRT2*, *MST1R* and *NNAT*) had a significant correlation for the hypomethylated samples (Table 3). Scatter plots of the representative CpG sites in the promoters of *DMRT2* and *NNAT* are detailed in Additional file 2: Figure S6.

Within the set of 35 CpG sites, the highest inverse correlation was observed for three CpG sites in the promoter and gene body of *ALDH1A3*, a member of the aldehyde dehydrogenase 1 family. The location of CpG sites has been reported to influence the effect of DNA methylation on gene regulation. High levels of gene body methylation have been positively correlated with an increase in gene expression [6]. Two CpG sites of the positively correlated genes were located in the gene body. One was located in *SHANK2* and the second between exons 2 and 3 of *CDKN2A* (Additional file 2: Figure S8). Positive correlations between gene expression and DNA methylation of a CpG site in the promoter were observed for *SOX7*, *SHANK2*, and *MICALL2* (Table 3).

### Identification of a minimal differential set

**Figure 6:**
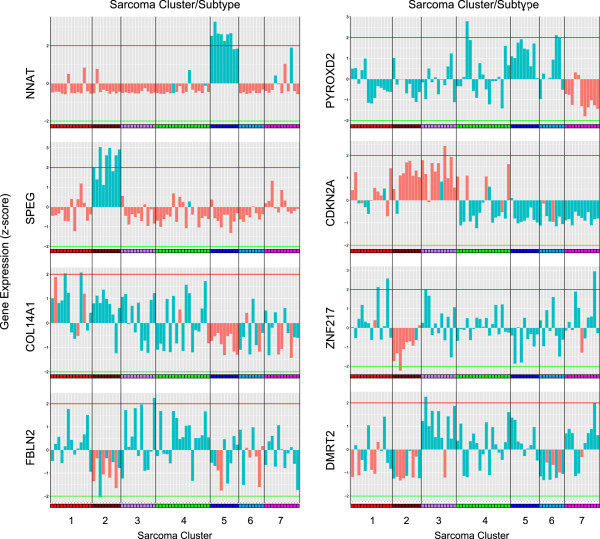
**The minimal differential set.** DNA methylation and gene expression profiles of the minimal differential set for discrimination of the seven sarcoma clusters (Tables 4 and 5). Hypermethylation and hypomethylation are shown in red and blue, respectively.

A minimal differential set was selected to represent the best pairwise differences between the seven methylation clusters. For each pairwise comparison of the sarcoma clusters one representative CpG was selected from the set of 35 CpG sites for which the methylation profiles showed a significant correlation with gene expression and the methylation level differed significantly between classes (Figure 5b). For each of the genes only significant pairwise differences between clusters were considered for the selection of the minimal differential set (*P* ≤ 0.05, pairwise Wilcoxon rank sum test with Benjamini–Hochberg correction for multiple testing over all pairwise comparisons). If a specific pairwise comparison was considered significant for multiple genes, the one with the highest area under curve (AUC) was selected. If multiple genes showed the same AUC, we selected the one ranked most informative by the Boruta method. Finally, the minimal differential set was composed of eight CpG sites and their corresponding genes: *SPEG*, *NNAT*, *FBLN2*, *PYROXD2*, *COL14A1*, *DMRT2*, *ZNF217* and *CDKN2A* (Tables 4 and 5, Figure 5, Additional file 2: Figures S5 and S6). The binarized DNA methylation status of the eight CpG sites together with the gene expression levels of the corresponding genes is detailed in Figure 5 for every sample in the sarcoma collection. The DNA methylation status of a CpG site within the promoter of *PYROXD2* was the most reliable differentiation marker for sarcoma cluster 7 (mainly SS samples) and could be used as a unique marker for the pairwise comparison of sarcoma cluster 7 with all other sarcoma clusters. *PYROXD2* is exclusively hypermethylated in SS samples and showed the highest importance of all preselected 216 CpG sites (Figures 4 and 6).

**Table 4:** Characteristics of the minimal CpG differential set for discrimination of the seven sarcoma clusters

**Cluster comparison(a versus b)**	**Short name**	**CpG**	**Gene symbol**	**Area under curve (*M *** **value) (%)**	**Hypermethylation (binary) (%)**		**Pairwise Wilcoxon test *P *** **value**	**Kruskal–Wallis *P *** **value**	**CpG region**
					**Cluster a**	**Cluster b**			
1–2	4-02	cg10062065	*SPEG*	100	0	100	4.7 × 10^–5^	7.3 × 10^–4^	P
1–3	1-02	cg16907566	*COL14A1*	90	0	23	1.2E-03	9.6 × 10^–5^	P
1–4	3-30	cg00250430	*DMRT2*	85	0	61	2.3E-03	1.2 × 10^–4^	P
1–5	1-01	cg22298088	*NNAT*	100	0	100	1.8 × 10^–5^	1.1 × 10^–5^	P
1–6	5-01	cg00201234	*FBLN2*	100	67	0	4.2 × 10^–5^	1.1 × 10^–4^	P
1–7	2-01	cg08397758	*PYROXD2*	100	100	0	8.4 × 10^–6^	3.2 × 10^–4^	P
2–3	4-02	cg10062065	*SPEG*	100	100	0	6.3 × 10^–5^	7.3 × 10^–4^	P
2–4	4-06	cg00476577	*ZNF217*	99	0	89	2.7 × 10^–5^	1.9 × 10^–4^	P
2–5	4-02	cg10062065	*SPEG*	100	100	0	9.1 × 10^–5^	7.3 × 10^–4^	P
2–6	4-02	cg10062065	*SPEG*	100	100	0	1.4 × 10^–4^	7.3 × 10^–4^	P
2–7	2-01	cg08397758	*PYROXD2*	100	100	0	2.9 × 10^–4^	3.2 × 10^–4^	P
3–4	3-07	cg10895543	*CDKN2A*	91	29	91	4.7 × 10^–5^	2.8 × 10^–6^	GB
3–5	1-01	cg22298088	*NNAT*	100	0	100	3.0 × 10^–5^	1.1 × 10^–5^	P
3–6	5-01	cg00201234	*FBLN2*	100	67	0	8.3 × 10^–5^	1.1 × 10^–4^	P
3–7	2-01	cg08397758	*PYROXD2*	100	100	0	2.0 × 10^–5^	3.2 × 10^–4^	P
4–5	1-01	cg22298088	*NNAT*	100	0	82	5.0 × 10^–6^	1.1 × 10^–5^	P
4–6	5-01	cg00201234	*FBLN2*	99	67	0	2.7 × 10^–5^	1.1 × 10^–4^	P
4–7	2-01	cg08397758	*PYROXD2*	100	100	0	2.0 × 10^–6^	3.2 × 10^–4^	P
5–6	1-01	cg22298088	*NNAT*	100	0	100	7.6 × 10^–5^	1.1 × 10^–5^	P
5–7	2-01	cg08397758	*PYROXD2*	100	100	0	3.0 × 10^–5^	3.2 × 10^–4^	P
6–7	2-01	cg08397758	*PYROXD2*	100	100	0	4.2 × 10^–5^	3.2 × 10^–4^	P

GB, gene body; P: promoter.

**Table 5:** Functional annotation of the minimal CpG differential set for discrimination of the seven sarcoma clusters

**Cluster comparison**	**Short name**	**CpG**	**Gene symbol**	**Gene name**	**Functional annotation**^**a**^
1-7; 2–7; 3–7; 4–7; 5–7; 6-7	2-01	cg08397758	*PYROXD2*	Pyridine nucleotide-disulfide oxidoreductase domain 2	Oxidoreductase activity
1-2; 2–3; 2–5; 2-6	4-02	cg10062065	*SPEG*	Striated muscle preferentially expressed protein kinase	Muscle organ development, regulation of cell proliferation, muscle cell differentiation
1-5; 3–5; 4–5; 5-6	1-01	cg22298088	*NNAT*	Neuronatin	Regulation of peptide secretion, neuron differentiation, regulation of protein localization
1-6; 3–6; 4-6	5-01	cg00201234	*FBLN2*	Fibulin 2	Regulation of cell–substrate adhesion, extracellular matrix binding
1-3	1-02	cg16907566	*COL14A1*	Collagen, type XIV, alpha 1	Cell–cell adhesion, extracellular matrix organization
1-4	3-30	cg00250430	*DMRT2*	Doublesex and mab-3 related transcription factor 2	DNA-dependent transcription
2-4	4-06	cg00476577	*ZNF217*	Zinc finger protein 217	Regulation of transcription
3-4	3-07	cg10895543	*CDKN2A*	Cyclin-dependent kinase inhibitor 2A (melanoma, p16, inhibits CDK4)	Cell cycle arrest, induction of apoptosis, negative regulation of cell proliferation

^a^Functional annotation from Gene Ontology website [37].

The top markers for differentiation of sarcoma cluster 2 (LMS samples) and sarcoma cluster 5 (MLS samples) were *SPEG* (*striated muscle preferentially expressed protein kinase*) and *NNAT* (*neuronatin*), respectively. Both genes are hypomethylated and highly expressed mainly in LMS and MLS samples, respectively. A CpG site in the promoter of *fibulin 2* (*FBLN2*) was the most important marker for sarcoma cluster 6 (MPNST samples) compared to sarcoma clusters 1, 3 and 6 (mainly consisting of DDLS, PLS and, MFS/UPS samples). A member of the collagen family (*COL14A1*) was selected to distinguish between sarcoma cluster 1 (mainly DDLS samples) and sarcoma cluster 3 (mainly PLS samples).

As differentiation markers for the UPS/MFS cluster (sarcoma cluster 4) *DMRT2*, *ZNF217* and *CDKN2A* were selected for comparison with sarcoma cluster 1 (mainly DDLS samples), sarcoma cluster 2 (LMS samples) and sarcoma cluster 4 (mainly PLS samples), respectively. The AUC reached 85%, 99% and 91%, respectively. The differentiation performance translates to hypo- and hypermethylation status as visualized in Additional file 2: Figure S5.

### Identification of liposarcoma-specific CpG sites

To identify histology-specific CpG sites important for liposarcoma pathogenesis and progression, we compared the DNA methylation status of each liposarcoma subtype with two normal, non-neoplastic fat samples. CpGs were filtered to identify those that showed an especially high change in DNA methylation. For this, the cluster-wise mean of each probe’s DNA methylation level was calculated and divided by the respective methylation level of a fat sample. This was repeated for each of the two fat samples. Only if a fold change above the 95% quantile (or below the 5% quantile) was observed for both fat samples was the change in DNA methylation level considered stable. The same procedure was also applied to the gene expression data (Table 6, Additional file 1: Tables S10 and S11). A CpG site was considered as differential between fat and a given liposarcoma subgroup if these criteria were met for both DNA methylation and gene expression. Additionally, the relation between respective DNA methylation and gene expression changes had to show the same direction as the correlation (positive/negative) detected over all primary sarcoma samples (Table 3 and Additional file 1: Table S7).

**Table 6:** CpG sites in liposarcoma subtypes

**Short name**	**CpG site**	**Gene symbol**	**FC methylation**	**FC expression**
**Cluster 1 (DDLS) versus fat**				
3–07	cg10895543	*CDKN2A*	13.1	9.2
**Cluster 3 (PLS) versus fat**				
3–07	cg10895543	*CDKN2A*	27.7	11.2
3–13	cg27652350	*ALDH1A3*	1/7.7	1/7.6
**Cluster 5 (MLS) versus fat**				
1–01	cg22298088	*NNAT*	1/9.8	6.0
1–13	cg12862537	*NNAT*	1/13.3	6.0
1–03	cg18508525	*CD36*	1/19.8	7.2
1–11	cg25181284	*GPD1*	1/9.1	1/2.4
1–14	cg04396791	*SHANK2*	19.9	3.3
1–18	cg10362475	*SHANK2*	8.1	3.3
1–20	cg01473816	*ELF5*	1/5.8	4.0
1–26	cg21359747	*ALDH1A3*	33.0	1/34.8
3–13	cg27652350	*ALDH1A3*	8.0	1/34.8
3–38	cg20786074	*EFEMP1*	9.4	1/22.5

DDLS, dedifferentiated liposarcoma; MLS, myxoid liposarcoma; PLS, pleomorphic liposarcoma.

FC is defined as “fold change”.

Meeting these criteria, we observed hypermethylation of a CpG site located in the gene body of the tumor-suppressor gene *CDKN2A* in cluster 1 (DDLS) and cluster 3 (PLS) compared to both normal fat samples (CpG short name 3–07 in Additional file 2: Figure S7a, b). A strong positive relation between methylation and gene expression for *CDKN2A* was identified for both clusters. As mentioned above, this CpG site had a positive correlation between methylation and gene expression for the whole sarcoma collection. The CpG site is located in the neighborhood of a predicted CpG island in the gene body of *CDKN2A* (between exons 2 and 3; Additional file 2: Figure S8a).

For MLS (cluster 5), we found ten CpG sites that met these criteria (Table 6, Additional file 2: Figure S7c). *NNAT*, *CD36* and *ELF5* were hypomethylated and *ALDH1A3* and *EFEMP1* were hypermethylated in MLS samples. All these genes had an inverse correlation between DNA methylation and gene expression. *GPD1* was hypomethylated and *SHANK2* was hypermethylated in MLS samples; however, both were positively correlated with gene expression.

*ALDH1A3* had the highest negative correlation between DNA methylation and gene expression in the whole sarcoma collection and was downregulated in MLS samples compared to normal fat. Thus, we validated both the expression and methylation of *ALDH1A3*. Using pyrosequencing, the mean level of methylation in the first intron of *ALDH1A3* in six non-neoplastic fat samples was found to be 23.5% compared to 73.6% in nine MLS samples from the sarcoma collection (Additional file 2: Figure S9d,e). A quantitative PCR analysis showed that the expression of *ALDH1A3* was nearly undetectable in MLS compared to non-neoplastic fat (Additional file 2: Figure S9f).

### DNA methylation patterns in sarcoma cell lines

**Figure 7:**
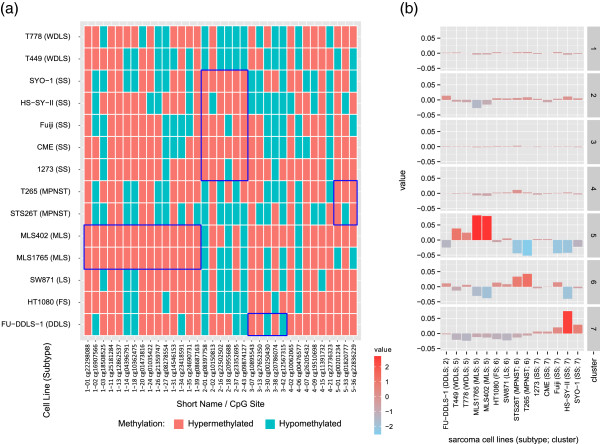
**Binarized methylation status in sarcoma cell lines. (a)** Binarized methylation status of the selected 35 CpG sites from the primary sarcoma collection in 14 sarcoma cell lines representing different sarcoma subtypes. Cluster-specific CpG sites are bordered in blue for the respective cell lines from which they were originally established. **(b)** Similarity of the sarcoma cell lines with the seven clusters of primary sarcomas. Both MLS cell lines (MLS402 and MLS1765) and both MPNST cell lines (STS26T and T265) show a significantly higher DNA methylation pattern similarity to their respective primary sarcoma subtypes than any of the other cell lines (*P* = 0.02 for MLS and MPNST cell lines). The five SS cell lines show a significantly higher methylation pattern similarity to primary SS samples (*P* = 0.001) than the remaining cell lines. DDLS, dedifferentiated liposarcoma; FS, fibrosarcoma; LS, liposarcoma; MLS, myxoid liposarcoma; MPNST, malignant peripheral nerve sheath tumor; SS, synovial sarcoma; WDLS, well-differentiated liposarcoma.

A significant part of our knowledge of the pathogenesis of soft tissue sarcomas is based on *in vitro* studies using sarcoma-derived cell lines. These representative cell lines are indispensable for functional studies. Thus, we analyzed the binarized DNA methylation status of the 35 selected markers derived from the primary sarcoma collection for 14 sarcoma cell lines representing different sarcoma subtypes. Binary values were used, as they offer a better translatability and interpretability between primary samples and cell lines. We observed that almost all markers for CpG cluster 1 (MLS samples) and for CpG cluster 2 (SS samples) showed an enhanced hypermethylated phenotype (Figure 7a and Additional file 2: Figure S4b).

In a second approach, we applied an RF model trained on binary data from the primary sarcomas on the sarcoma cell lines. For this RF classifier, we used only CpG sites that were selected as informative by Boruta. This model was applied to the binarized methylation data of the cell lines. The percentage of trees voting for a specific class (cluster) was used as a similarity measure for the cell lines compared to the identified primary sarcoma clusters. The raw votes (uncentered) and the cluster-wise mean centered votes are reported in Additional file 1: Table S12 and Figure 7b. Here, the methylation pattern of the two MLS cell lines (MLS402 and MLS1765) showed a significantly higher similarity to the methylation pattern observed in primary MLS samples (*P* = 0.02, Wilcoxon rank sum test) than the other cell lines. The two WDLS cell lines (T449 and T778) showed an increased similarity with primary MLS samples (cluster 5, *P* = 0.2, Wilcoxon rank sum test). The two MPNST cell lines (STS26T and T265) showed a significant increased similarity to the MPNST methylation cluster (cluster 6, *P* = 0.02, Wilcoxon rank sum test) than any of the other cell lines. According to the RF model, the five SS cell lines showed a significantly higher degree of similarity with cluster 7 (*P* = 0.001, Wilcoxon rank sum test). Of these, Fuji, HS-SY-II, and SYO-1 were the most similar. The DDLS cell line FU-DDLS-1, the fibrosarcoma cell line HT1080 and the liposarcoma cell line SW872 did not show a significantly higher degree of similarity with cluster 5 (*P* = 0.44, Wilcoxon rank sum test) than the remaining cell lines.

### Functional analysis of genes from the myxoid liposarcoma CpG cluster

**Figure 8:**
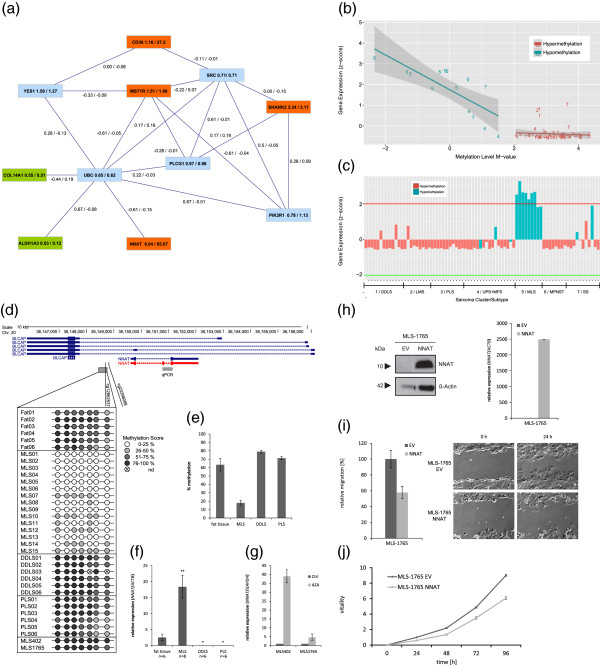
**Functional validation of *neuronatin *(*NNAT*). (a)** Gene network through interaction analysis of the genes from the MLS CpG cluster. Connections represent known protein-wise interactions. Indicated are genes that show differential DNA methylation and upregulation (red) or downregulation (green) in MLS compared to the other CpG clusters. Numbers on the solid lines represent correlation of gene expression (Kendall’s tau) within the MLS samples and across the remaining sarcoma samples. Numbers after the gene name are the gene expression fold change compared to the normal fat samples and the remaining sarcoma samples. **(b)** Correlation between *NNAT* expression and DNA methylation of CpG cg22298088 for the whole sarcoma collection. The numbers of the sample clusters are shown. The solid line represents the correlation trend (Kendall’s tau -0.303; *P* = 0.001). **(c)***NNAT* expression for the sarcoma collection together with binarized methylation status (*P* = 1 × 10^–5^; expression fold change 24.7; methylation fold change 9.3). **(d)** Position of seven CpGs in the direct neighborhood of cg12862537 and cg22298088. The methylation levels were analyzed in fat tissue, three liposarcoma subtypes and two MLS cell lines. **(e)** Mean level of DNA methylation of all eight CpGs shown in **(d)** for fat tissue and the three liposarcoma subtypes and **(f)** validation of *NNAT* expression (red transcript in (d)). **(g)** Re-expression of *NNAT* in MLS cell lines MLS402 and MLS1765 following 5-aza-dC treatment. **(h)** Western blot and quantitative PCR analysis after stable *NNAT* re-expression in MLS1765. Recovery of *NNAT* caused **(i)** decreased migration revealed by wound-healing assay after 24 h compared to the empty vector cell line (an illustrative example from three independent experiments is shown) and **(j)** diminished cell proliferation determined by the 3-(4,5-dimethylthiazol-2-yl)-2,5-diphenyltetrazolium bromide assay. Error bars represent standard error of the mean (*t*-test, * indicates *P* ≤ 0.05, ** indicates *P* ≤ 0.01). 5-aza-dC, 5-aza-2-deoxycytidine; Chr: chromosome; Ctrl, control; DDLS, dedifferentiated liposarcoma; EV, empty vector; LMS, leiomyosarcoma; MFS, myxofibrosarcoma; MLS, myxoid liposarcoma; MPNST, malignant peripheral nerve sheath tumor; PLS, pleomorphic liposarcoma; nd, not determined; SS, synovial sarcoma; UPS, undifferentiated pleomorphic sarcoma.

As MLS formed a very stable cluster and showed consistent changes compared to non-neoplastic fat, we decided to perform a functional analysis of our MLS related results. For this, we carried out a BisoGenet-based query of interaction database analyses [38] for the 12 genes from the MLS CpG cluster that have a reliable influence on gene expression. Linker genes were only considered if they directly connected two of these relevant genes [39]. The most valid network considered six of the twelve relevant genes (*NNAT*, *ALDH1A3*, *COL14A1*, *MST1R*, *CD36* and *SHANK2*) and five linker genes (*UBC*, *YES1*, *PLCG1*, *PIK3R1* and *SRC*). Ubiquitin C (*UBC*) was highly connected in the network and is known to show direct protein-wise interactions with *NNAT*, *COL14A1*, *ALDH1A3* and *MST1R* (Figure 8a, Additional file 2: Figure S9b). *UBC* was found to be significantly downregulated in primary MLS samples (*P* = 5.794 × 10^–5^, Wilcoxon rank sum test). For most of the interaction partners a stronger correlation (Kendall correlation) in gene expression was observed within the MLS samples than within the remaining primary sarcoma samples. For all 12 relevant genes from the MLS-associated CpG clusters, the gene-wise correlations (Kendall correlation) were considerably stronger within the MLS samples than within the other primary sarcoma samples (Additional file 2: Figure S9a). A second hub node was the tyrosine-protein kinase SRC, which is known for protein-wise interactions with *CD36*, *MST1R* and *SHANK2* (Figure 8a).

### Functional validation of *NNAT* from the myxoid liposarcoma CpG cluster

Of the 12 genes from the MLS CpG cluster, *NNAT* showed the highest and most significant changes in DNA methylation and inverse gene expression compared to non-neoplastic fat and the other sarcoma samples (Tables 3 and 6, Figure 8b,c). Furthermore, the CpG site cg22298088 in the promoter region of *NNAT* had the highest importance for classification of MLS in the whole sarcoma collection (Figures 4 and 6; Table 2). To validate changes in methylation status and gene expression of *NNAT*, we carried out pyrosequencing and qPCR in three subtypes of liposarcomas (DDLS, PLS and MLS) and normal fat samples (Figure 8d, e, f). *NNAT* is located within the first intron of *BLCAP* on chromosome 20q11.23 (Figure 8d). Pyrosequencing of eight CpG sites in the direct neighborhood of the CpG site cg22298088 verified the hypomethylation of *NNAT* in MLS compared to DDLS and PLS. The methylation frequency for *NNAT* in the six normal fat samples was 63.4%. In spite of the high methylation frequency, the fat samples showed consistently high *NNAT* expression levels compared to DDLS and PLS samples, which showed only a 15.6% and 7.9% higher methylation frequency, respectively, but almost an entire loss of *NNAT* expression. On the other hand, the average DNA methylation frequency in MLS was about 45% lower compared to the six non-neoplastic fat samples and was accompanied by a higher *NNAT* expression (Figure 8e,f). The two MLS cell lines, MLS402 and MLS1765, had a high degree of DNA methylation and absent *NNAT* expression. Treatment of the two cell lines with the demethylating agent 5-aza-2-deoxycytidine (5-aza-dC) reactivated the *NNAT* expression in MLS402 but only marginally in MLS1765 (Figure 8g). To evaluate the functional relevance of *NNAT*, we stably reconstituted *NNAT* expression in the hypermethylated cell line MLS1765 (Figure 8h). We observed that stable expression of *NNAT* caused a significant reduction in the migration rate (Figure 8i) and decreased cell proliferation in the myxoid liposarcoma cell line MLS1765 (Figure 8j). Apoptosis was not affected by overexpression of *NNAT* (Additional file 2: Figure S9c).

## Discussion

Several studies have shown that changes in DNA methylation for a growing number of genes play an essential role in cancer development, emphasizing the crucial role of these epigenetic changes for future diagnosis, prognosis and prediction of response to therapies [40,41]. Previous studies of epigenetic alterations in soft tissue sarcomas either focused on specific candidate genes or particular sarcoma subtypes [26,32,42,43]. In the current study, we simultaneously considered DNA methylation status and gene expression levels in a large and representative cohort of 80 untreated, primary high-grade sarcomas composed of eight subtypes to identify new candidate genes and to discriminate the different subtypes. In the unsupervised clustering, the two translocation-associated sarcoma subtypes, MLS and SS, formed two clusters according to their histopathological classification. This corresponds to unsupervised hierarchical cluster analyses in previous mRNA and miRNA expression studies, in which there was tight clustering of translocation-associated sarcoma samples whereas sarcoma samples with complex karyotypes tended to form more dispersed and heterogeneous clusters [23,44]. Using a random forest clustering approach that integrated histopathological groupings, we identified seven stable sarcoma subgroups of which five were associated with distinct DNA methylation clusters. The remaining three sarcoma clusters were defined by different multivariate methylation patterns of the same methylation cluster. Based on our DNA methylation data, most of the MFS and UPS samples had a similar DNA methylation pattern and formed one common sarcoma cluster. It is proposed that myxofibrosarcomas are myxoid variants of UPS. Both subtypes are characterized by frequent and complex genetic rearrangements; however, no chromosomal aberrations specific to UPS or MFS have been identified so far [15,45].

Using the DNA methylation status of a CpG site in the promoter of *PYROXD2*, a putative pyridine nucleotide-disulfide oxidoreductase gene with an uncharacterized functional role, we were able to separate the entire cluster 7 from the whole sarcoma collection with an AUC of 100%. This CpG site was hypermethylated exclusively in samples of sarcoma cluster 7, which comprised all SS samples and one MPNST sample. This MPNST sample had a DNA methylation pattern highly similar to the SS cluster. Of interest, this is the same MPNST sample that had a miRNA pattern highly similar to SS samples but for which initial diagnosis could be confirmed by histological re-evaluation and the absence of a *SS18-SSX1/2* fusion transcript (Renner *et al*. [23]). Distinguishing SS from MPNST can be challenging because of overlapping histologic features and immunohistochemical reactivity patterns of several markers [46-48]. A comparative methylome analysis of benign and malignant peripheral nerve sheath tumors was able to discriminate between disease phenotypes [42]. The detection of alterations in DNA methylation is a promising tool for the diagnosis and prognosis of disease [40,49]. Compared to protein-based analysis, the DNA methylation status of *ZAP-70* provides more accurate prognostic information for chronic lymphocytic leukemia [50]. The DNA methylation of *MGMT* has been found to be a more reliable predictor of outcomes in glioblastoma patients [51]. Therefore, the differential DNA methylation status of just one CpG site in the promoter of *PYROXD2* may help differentiation between SS and MPNST or the other sarcoma subtypes analyzed in this study*.* Interestingly, this CpG site in the promoter of *PYROXD2* also perfectly separates SS cell lines from the remaining cell lines. Further investigation using an independent cohort involving a large number of patients with SS or MPNST is needed to assess the relevance of this CpG site as a diagnostic marker for these kinds of sarcoma subtypes.

The top marker for differentiation between sarcoma cluster 3, which is mainly composed of PLS samples, and sarcoma cluster 4, which is composed of MFS and UPS samples, was a CpG site located in the gene body of *CDKN2A*. Furthermore, we identified a correlation between gene expression and DNA methylation of this CpG site in PLS and DDLS compared to non-neoplastic fat tissue. However, the DNA methylation status of this CpG site was positively correlated with *CDKN2A* gene expression. Several studies have described and discussed high levels of intragenic (gene body) DNA methylation and increased gene expression [6,52,53]. The p16^INK4A^ protein product of the *CDKN2A* locus is known to be an important tumor-suppressor gene, which directly inhibits the kinases encoded by the oncogenes *CDK4* and *CDK6*[54,55]. The *CDKN2A* locus on chromosome 9p21 is frequently mutated or deleted in a variety of carcinomas as well as in soft tissue sarcomas [56-62]. However, hypermethylation of the promoter region of *CDKN2A* seems to have only a limited effect on gene inactivation [56,60]. To our knowledge, this is the first report of differential *CDKN2A* expression between UPS/MFS and PLS and of DNA methylation in the gene body as a potential regulator of *CDKN2A* expression. Further investigation of the identified CpG site in the gene body of *CDKN2A* in combination with genetic alterations for a large cohort of these sarcoma subtypes may identify a new diagnostic option for stratification of high-grade pleomorphic sarcomas.

A CpG site within the promoter of *fibulin 2* (*FBLN2*) was identified as discriminating for MPNST samples (sarcoma cluster 6) versus sarcoma clusters 1, 3 and 4. *FBLN2* encodes for a member of the fibulin family of extracellular matrix proteins, which interact with various extracellular ligands. *FBLN2* is hypermethylated in breast cancer and has a tumor-suppressive role in nasopharyngeal carcinomas [63,64]. Further genes in the minimal differentiation set were *ZNF217*, *COL14A1* and *DMRT2. ZNF217* is a marker of poor prognosis in breast cancer [65], *COL14A1* is a candidate tumor-suppressor gene frequently methylated in renal cell carcinomas [66] and the transcription factor *DMRT2* is downregulated in clear-cell renal-cell carcinomas [67].

In general, we observed a tendency for a higher DNA methylation status of subgroup-specific CpG sites in sarcoma cell lines than in the respective primary sarcomas. The higher frequency of hypermethylation might be a consequence of the accumulation of epigenetic changes during prolonged cell culture. These findings are consistent with reports describing significant differences in DNA methylation and gene expression between cancer cell lines and tumors of several entities [13,68,69]. This indicates that sarcoma cell lines are useful for molecular and epigenetic studies, especially for hypermethylated genes (for example, *ALDH1A3*) but are only of limited use for hypomethylated genes. The methylation data for the sarcoma cell lines provide a basis for selective use of these cell lines for further basic and translational research with respect to their DNA methylation environment.

To identify DNA methylation changes in sarcomas that are of functional relevance, we integrated DNA methylation and mRNA expression data. A strong negative correlation was observed between DNA methylation status and expression of *ALDH1A3* for the whole sarcoma collection. Furthermore, *ALDH1A3* was the most hypermethylated and downregulated gene for MLS compared to normal fat. *ALDH1A3* is a member of the aldehyde dehydrogenase family with 19 isoenzymes, which are thought to play a major role in the detoxification of aldehydes generated by alcohol metabolism and lipoid peroxidation [70]. Recently, it has been reported that *ALDH1A3* can function as a novel marker of cancer stem cells and predict clinical prognosis in breast cancer and glioblastoma [70,71]. In a liposarcoma xenograft model, a small population of *ALDH1A1*- and *CD133*-expressing cells had inducible cancer stem cell potential [72], and high ALDH1 activity in sarcoma cell lines was characterized by a significantly increased proliferation rate [73].

Another gene that was hypermethylated and downregulated in MLS compared to normal fat was *EFEMP1.* This gene encodes fibulin-3, a member of the fibulin family. These proteins are extracellular matrix glycoproteins with repeated epidermal growth factor-like domains [74]. In cervical carcinomas, *EFEMP1* promotes angiogenesis, accelerates tumor growth *in vivo* and is associated with lymph node metastasis, vascular invasion and poor prognosis [75,76]. Recently, fibulin-3 was identified as a blood and effusion biomarker for pleural mesothelioma [77]. Downregulation of *EFEMP1* was closely associated with promoter hypermethylation in breast, hepatocellular, colorectal, prostate and non-small cell lung carcinomas [78-83]. Based on our data, *EFEMP1* is possibly a tumor suppressor in several types of cancer including MLS. On the other hand, *EFEMP1* promoted tumor growth in pancreatic adenocarcinomas and acted as an oncogene [84].

The top marker for identification of LMS samples, which showed significant correlation between DNA methylation and gene expression, was *SPEG*, which was originally found to be preferentially expressed in differentiated vascular smooth muscle cells [85]. In the whole sarcoma collection, *SPEG* was hypomethylated and highly expressed exclusively in LMS samples. Indeed, LMS is the only subtype within the sarcoma collection that shows smooth muscle differentiation. The gene product of *SPEG* is similar to members of the myosin light chain kinase family and is thought to be a differentiation marker for smooth muscle.

In the whole sarcoma collection, we identified a significant correlation between gene expression and DNA methylation of two CpG sites in the promoter of *Neuronatin* (*NNAT*). One of the two CpG sites was the most important differentiation marker for MLS. Furthermore, *NNAT* was one of the top hypomethylated and upregulated genes in the comparison of normal fat samples and MLS. *NNAT* is imprinted and actively transcribed exclusively from the paternally inherited allele. Originally, *NNAT* was identified as a brain-specific gene expressed during brain and pituitary development [86-88]. Regulation of *NNAT* expression by DNA methylation was first described for pituitary adenomas and later for pediatric acute leukemias [89,90]. *NNAT* is located on chromosome 20q11.2 and resides within an intron of the non-imprinted gene *Bladder Cancer-Associated Protein* (*BLCAP*) [91]. It was hypothesized that reactivation of maternal *NNAT* would lead to an overall downregulation of *BLCAP*[92]*.* However, the DNA methylation status of the *BLCAP* promoter was not significantly different in our sarcoma collection, and *BLCAP* had homogeneous high expression levels.

In the context of our findings, it is of interest that *NNAT* was previously reported to be upregulated in MLS compared to normal fat [93] and that ectopic expression of *NNAT* in pre-adipocytes stimulated differentiation into mature adipocytes by induction of adipogenic transcription factors [94]. Compared to normal fat samples we found DNA hypomethylation and high expression of *NNAT* in MLSs. On the other hand, we observed DNA hypermethylation and complete downregulation of *NNAT* in two further liposarcoma subtypes, namely DDLS and PLS, indicating hampered or complete disruption of normal adipogenesis in these subtypes. In MLS, demethylation and reactivation of the maternal *NNAT* allele may have occurred. On the other hand, *de novo* methylation of the unmethylated paternal allele of *NNAT* may have occurred in DDLSs and PLSs, a process described as loss of imprinting [95]. Stable reconstitution of *NNAT* in the hypermethylated cell line MLS1765 caused decreased cell proliferation and reduced cell migration, matching the criteria for a putative tumor-suppressor gene. The subclassification of liposarcomas has important prognostic significance: patients with pleomorphic and dedifferentiated liposarcomas have an unfavorable prognosis compared to patients with MLS or WDLS [96,97]. However, in contrast to our data identifying *NNAT* as a putative tumor suppressor in MLS, it was recently shown that high *NNAT* expression correlates with decreased survival of patients with glioblastoma [98], and that silencing of *NNAT* through *miR-708* promotes cell migration and metastasis formation in breast cancer [99]. Since *miR-708* is not differentially expressed in the liposarcoma samples of our collection [23], DNA methylation seems to be the predominant mechanism for the regulation of *NNAT* expression in liposarcomas.

## Conclusions

In summary, our DNA methylation and gene expression approach for a collection of 80 primary, high-grade soft tissue sarcomas and 14 sarcoma cell lines, accomplished four aims: (1) the identification of diagnostically relevant DNA methylation differences between different sarcoma subtypes, (2) the identification of new subtype-specific and functionally relevant candidate genes that showed correlation between DNA methylation and gene expression, (3) the identification of DNA methylation patterns in sarcoma cell lines, which could be used in the future for the functional validation of candidate genes that show gene expression changes influenced by DNA methylation and (4) the identification of new and functionally relevant DNA methylation differences between liposarcomas and non-neoplastic fat tissue with *NNAT* as a new potential tumor-suppressor gene for MLS. It is essential to analyze whether the differentially methylated candidate genes identified in our study could be used to improve the diagnosis, prognosis and therapy of patients with soft tissue sarcomas.

## Materials and methods

### Clinical specimens

The sarcoma samples were collected at the Institute of Pathology, University of Heidelberg, snap-frozen in liquid nitrogen after surgical removal and stored at -80°C. The collection was composed of eight sarcoma subtypes: dedifferentiated liposarcomas (DDLSs), leiomyosarcomas (LMSs), myxofibrosarcomas (MFSs), malignant peripheral nerve sheet tumors (MPNSTs), myxoid liposarcomas (MLSs), pleomorphic liposarcomas (PLSs), synovial sarcomas (SSs) and undifferentiated pleomorphic sarcomas (UPSs, formerly called malignant fibrous histiocytomas).

Diagnoses were based on current standard histopathological criteria in conjunction with immunohistopathological and molecular analysis according to the current WHO classification of tumors [15]. The lymphohistiocytic inflammatory stromal component was determined by immunohistochemistry using antibodies against CD3 (BD Biosciences, Heidelberg, Germany), CD20 and CD68 (Dako, Hamburg, Germany) on frozen sections. Only samples with low inflammatory stromal components that contained at least 80% vital tumor cells were selected for the analysis. Detection of fusion transcripts in MLS and SS samples and immunostaining for MDM2 and CDK4 in DDLS samples was carried out as described [23]. The study was approved by the local ethics committee (No. 206/2005, 207/2005). The patients’ characteristics are shown in Additional file 1: Table S1.

### Illumina Infinium methylation assay

The Infinium HumanMethylation27 BeadChip v1.2 system (Illumina, San Diego, CA) was used to obtain genome-wide DNA methylation profiles of 27,578 CpG dinucleotides located in a region of 1 kb around the transcription start site of 14,495 genes [100]. Genomic DNA was isolated using the Allprep DNA/RNA Mini Kit (Qiagen, Hilden, Germany) followed by ethanol precipitation with 5 M ammonium acetate. Bisulfite conversion was carried out using the EZ DNA Methylation Kit (Zymo Research, Irvine, USA) according to the manufacturer’s instructions and 500 ng of the bisulfite-converted genomic DNA was used with the Infinium bead array platform. All samples were tested in the Core Facility of the German Cancer Research Center (DKFZ), Heidelberg. The methylation status obtained from this assay was expressed as the ratio of fluorescence intensity of the methylated probe over the overall intensity (beta value) and the log_2_ ratio of the intensities of the methylated probe versus the unmethylated probe (*M* value) [101]. If not specified otherwise, the *M* values were used for all statistical tests, model construction and visualization. Based on these *M* values obtained from the probe intensities, a partitioning algorithm was adapted to classify each sample’s methylation status [33]. The methylation status of each sample at a given locus was binarized as 1 (hypermethylated) or 0 (hypomethylated). Probes for which the algorithm was not able to binarize the intensities were removed from further analysis. A detailed description of the algorithm can be found in Supplemental document 1 in Additional file 2. An R implementation can be obtained from Additional web resource 1. The binarized matrix is referred to as binarized methylation, while the unbinarized *M* values are referred to as raw data.

### Gene expression assay

Quality control and quantification of total RNA were conducted using a RNA 6000 nano LabChip with an Agilent 2100 Bioanalyzer (Agilent Technologies, Palo Alto, CA). Only RNA with a RNA integrity number >7 was used for microarray-based mRNA profiling. Expression profiling was performed using the HumanHT-12 v3 BeadArrays (Illumina) according to the manufacturer’s instructions. Quality control as well as labeling and hybridization were performed in the Core Facility of the DKFZ, Heidelberg. Annotation and quantile normalization were performed using the lumi R package [102,103]. Mapping between probes on the mRNA microarray and CpG sites on the methylation array was performed using the GenomicRanges R package.

### Statistical analysis

All statistical tests and algorithmic modeling used the open-source software R [104]. The plots were generated using the gplots and ggplots2 [105] R packages. *P* ≤ 0.05 was considered significant. Random forest models were generated using the randomForest package. Feature selection was performed using the Boruta package, cross validation using the ipred package and the classifier was rated using the caret package. Cluster stability was assessed using the fpc package.

### Univariate analysis

The reported fold changes between two groups for a respective feature (DNA methylation level (*M* values) or gene expression) were given as unlogged differences between the means of two comparison groups [106]. For the beta values, the absolute difference between the means of both groups was reported. For all following tests and analysis steps, the *M* values were used. The significance of a change between two groups was tested using a Wilcoxon rank sum test. Differences between more than two groups were analyzed using the Kruskal–Wallis one-way analysis of variance. To identify which pairwise differences between groups for a probe were significant, we also performed a pairwise Wilcoxon rank sum test. For this pairwise Wilcoxon rank sum test, a correction for multiple testing was performed over all pairwise comparisons for each gene. The significance of the correlation was assessed using the Kendall rank correlation coefficient [107]. The univariate partitioning ability of a probe’s *M* value was rated according to its AUC (area under curve) [108,109]. The multiple pairwise tests were corrected using the Benjamini–Hochberg false discovery rate approach [110].

### Unsupervised clustering

A cluster analysis is often considered to be the first step in the analysis of high-throughput biological data sets. For unsupervised clustering we used the divisive analysis (DIANA) approach [34]. DIANA is a hierarchical clustering algorithm, which computes a divisive hierarchy instead of an agglomerative one. The Euclidean distance was used as the distance metric.

### Random forest classification

Supervised machine learning algorithms are able to learn the molecular patterns of histopathologically defined groups, and can thus be used to select the most important variables for discriminating between these groups of interest. The random forest (RF) algorithm was used for classification. The RF method is an ensemble classifier that uses a collection of decision trees. Each tree is constructed using a bootstrap subsample of the data. Class assignment for a sample is performed separately for each tree in the collection. The percentage of trees voting for the class of interest is used to define a degree of class membership between 0% and 100%. The final class assigned to a sample is determined by the majority vote (>50%). These percentages can also be used as similarity measure when comparing a sample to a class from the training set. At each iteration (bootstrap subsampling) of the RF construction, the data that were not part of the training subsample (out-of-the-bag data) are used to estimate the error rate. The average (mean) error over all iterations is commonly referred to as the out-of-the-bag (OOB) error. Accuracy, sensitivity and specificity were calculated based on a class assignment according to the majority vote. CpG site importance can be estimated using the mean decrease in accuracy. This gives the increase in OOB error when the OOB data for that CpG site are permuted while all others are left unchanged. This global variable importance generated by RF captures the classification impact of variables on all samples.

The R package Boruta was used to achieve a more stable ranking of feature importance and to select only informative variables (probes) [111]. This algorithm uses the importance returned by RF to find all variables that are informatively related with class assignment [112]. Features that were selected with a confidence of at least 0.95 by Boruta were considered as informative.

### Model validation

The predictive performance of a classification model was assessed using either the OOB error (if no class-based feature preselection was performed) or the average error over ten repeats of the class-stratified tenfold cross validation. To achieve an unbiased estimation, all steps using class information were included in the cross validation.

### Random forest model analysis I: clustering of samples

RF not only generates variable-related information such as variable importance measures, but also calculates the proximity between samples. The proximity between similar samples is high. In proximity calculations, all samples in the original data set are classified by the forest. The proximity between two samples is calculated as the number of times the two samples end up in the same terminal node of a tree, divided by the number of trees in the forest. For this study, only the OOB proximity was used, which is only claculated when both samples were not part of the training set for a tree. Clustering based on distance (1 – proximity) was conducted using the partitioning around medoids (PAM) algorithm. The optimal number of clusters for PAM was chosen using the average cluster stability [35].

### Random forest model analysis II: clustering of genes

In addition to the global variable importance, RF also calculates the local variable importance [113]. This gives an estimate of the importance of a variable in the classification of a single sample. Thus for each variable/sample combination, an importance value was estimated. The correlation between probes (Pearson’s correlation coefficient, *r*) was then calculated using their local importance instead of the *M* values. These probes were then clustered based on correlation distance (1 – *r*) using PAM.

### Workflow

An RF model was trained to distinguish between the eight histopathologically defined classes, using all probes with a distinct bimodal methylation pattern. The OOB distance (1 – proximity) returned by this model was used to regroup the samples into clusters defined by methylation pattern. This approach integrated the histopathological findings and methylation patterns. The discovered groups formed the basis for all further analysis steps.

Differences between these newly defined groups were analyzed using the Kruskal–Wallis one-way analysis of variance. Probes with significant differences between classes were chosen as input for the Boruta algorithm. The probes selected by Boruta with a confidence of at least 0.95 served as input to the final RF classifier, which was trained on the newly defined methylation clusters.

### Pyrosequencing

Bisulfite pyrosequencing was performed on PyroMark Q24 (Qiagen) according to standard protocols. Templates were amplified using the PyroMark PCR Kit (Qiagen). Primer pairs were designed with the PyroMark Assay Design SW 2.0 (Qiagen) and data were evaluated with Pyro Q-CpG 1.0.9 (Biotage). The primer sequences are listed in Additional file 1: Table S13.

### RNA isolation and quantification

RNA was isolated from snap-frozen tissue using the Allprep DNA/RNA Mini Kit (Qiagen) according to the manufacturer’s instructions. Then 1 μg of total RNA was reverse transcribed with the RevertAid™ H minus Reverse Transcriptase (Fermentas, St Leon-Rot, Germany) and analyzed using the RT cycler ABI PRISM 7300 (Applied Biosystems, Darmstadt, Germany) with Absolute SYBR Green ROX Mix (Abgene, Epsom, United Kingdom). All samples were run in triplicate and 10 ng cDNA (relative to the inserted total RNA) was used per reaction. Relative quantification was carried out using the Delta Delta Ct (ΔΔCt) method and *ACTB* as an endogenous control. The primer sequences are listed in Additional file 1: Table S13.

### Cell culture, 5-aza-2-deoxycytidine treatment and functional assays

Cell lines used for the analyses together with references, molecular confirmation and culture conditions are detailed in Additional file 1: Table S2. For array-based methylation profiling, cell lines were grown to 80% confluence and trypsinized for DNA isolation. For gene re-expression, MLS402 and MLS1765 were incubated with 10 μM of 5-aza-dC (Sigma Aldrich, Steinheim, Germany) for 96 h. The culture medium and 5-aza-dC were replaced every day. Cell viability was measured using the MTT (3-[4,5-dimethylthiazol-2-yl]-2,5-diphenyl-tetrazolium-bromide)-assay (Sigma Aldrich) at the indicated time points. For cell migration, cells were plated in triplicate into six-well plates (5 × 10^5^ cells/well), cultured in RPMI containing 10% FBS and grown to confluence. Cells were treated with mitomycin C (5 μg/ml RMPI without FCS, 3 h), two scratch wounds were generated per well using a sterile plastic 200-μl pipette tip and floating debris was removed by washing with PBS. Cells were incubated in a Live Cell Imaging System (Olympus, Hamburg, Germany) and monitored for 24 h. The wound-healed area was measured as the ratio of the occupied area to the total area using AxioVision (Zeiss, Jena, Germany). Tumor cell apoptosis was measured using fluorescence-activated cell sorting (FACS) analysis of propidium iodide-stained nuclei with a FACS-Calibur flow cytometer (Becton-Dickinson, Heidelberg, Germany). After preparation according to [114], measurements were acquired in Fl-2 in logarithmic mode and calculated by setting gates over the first three decades to detect apoptotic cells.

### Cloning and stable transfection of NNAT

*NNAT* cDNA (transcript 1, alpha isoform, NM_005386.2) was cloned into the pDEST26 mammalian expression vector (Life Technologies, Darmstadt, Germany) using the Gateway LR Clonase II Enzyme Mix (Life Technologies) and transfected into MLS1765 using Attractene (Qiagen). To select stably transfected clones, cells were supplemented with G418 (400 μg/ml). Single clones were analyzed for *NNAT* expression by qPCR and Western blotting 30 days after plating.

### Protein extraction and Western blot

Cell pellets were lysed with Cell Lysis Buffer (Cell Signaling/New England Biolabs, Frankfurt, Germany) containing a protease inhibitor cocktail (Roche, Mannheim, Germany). Proteins were quantified with the Bio-Rad Protein Assay (Bio-Rad Laboratories, Munich, Germany) and Western blotting was performed using an antibody specific for *NNAT* (Cat# ab27266, Abcam, Cambridge, UK).

### Data access

Genome-wide data sets of all sarcoma samples included in this study have been submitted to the Gene Expression Omnibus (GEO) [115] under accession number GSE52392.

## Abbreviations

5-aza-dC: 5-aza-2-deoxycytidine; AUC: Area under curve; DDLS: Dedifferentiated liposarcoma; DFKZ: German Cancer Research Center, DIANA, divisive analysis; EV: Empty vector; FACS: Fluorescence-activated cell sorting; FCS: Fetal calf serum; FS: Fibrosarcoma; GIST: Gastrointestinal stromal tumor; kb: Kilobase; LMS: Leiomyosarcoma; LS: Liposarcoma; MFS: Myxofibrosarcoma; miRNA: microRNA; MLS: Myxoid liposarcoma; MPNST: Malignant peripheral nerve sheath tumor; OOB: Out of the bag; PAM: Partitioning around medoids algorithm; PBS: Phosphate-buffered saline; PLS: Pleomorphic liposarcoma; RF: Random forest; SS: Synovial sarcoma; STS: Soft tissue sarcoma; UPS: Undifferentiated pleomorphic sarcoma; WDLS: Well-differentiated liposarcoma.

## Competing interests

The authors declare that they have no competing interests.

## Authors’ contributions

MR, TW, GM, PL and PS designed the study. MR and TW analyzed the data and wrote the manuscript. MR designed and performed the functional analysis. VE performed the apoptosis assays. WH, EW and RB contributed the sarcoma cell lines. HM, TW, EC, RE, VH and BB contributed to the computational analysis. VH made a significant contribution to the annotation. GM, IA, AU, BL, GE, TS and EKR collected and processed the fresh material. GM, PS, EW, WH and RB provided pathological guidance. GM, RB, HM and PS edited the manuscript. All authors read and approved the final manuscript.

## Supplementary Material

Additional file 1: Table S1 Patient characteristics. Summary of clinical and molecular characteristics of the 80 primary sarcoma samples analyzed (NA, not available). **Table S2.** Cell line characteristics. Summary of the sarcoma cell lines analyzed in this study. (ATCC, American-type culture collection; PS, penicillin/streptomycin). **Table S3.** Classification performance. **Table S4.** Bootstrapping. **Table S5.** PAM classification. **Table S6.** Set of 216 CpG sites. **Table S7.** Beta values of the selected set/all CpGs. **Table S8.** Correlation with gene expression. **Table S9.** Cluster comparison. **Table S10.** Comparison of cluster versus fat methylation. **Table S11.** Comparison of cluster versus fat gene expression. **Table S12.** Cell line classification. **Table S13.** Primer sequences for pyrosequencing and qPCR. (Btn, biotin). Click here for file

Additional file 2: Figure S1 Methylation-based regrouping of histopathological sarcoma subtypes. **Figure S2.** Workflow cross validation. **Figure S3.** Methylation status of the two CpG sites located on the X chromosome ((a) M-values, (b) binarized). (c) Importance of the CpG subgroups for classifying of the sarcoma subgroups. **Figure S4.** Heatmap of selected markers in the (a) primary collection and in (b)sarcoma cell lines. **Figure S5.** Bar plots of the DNA methylation of the minimal differential set between the different sarcoma clusters. **Figure S6.** Scatter plots of the minimal differential set. **Figure S7.** Comparison of (a) cluster 1 (DDLS), (b) cluster 3 (PLS) and (c) cluster 5 (MLS) versus fat. **Figure S8.** (a) Position of the CpG site and CpG islands in *CDKN2A*, (b and c) DNA methylation and gene expression profile of *CDKN2A* in the whole sarcoma collection. **Figure S9.** (a) Correlation between *NNAT* and UBC, (b) PubMed identifier (PMID) of the protein-wise interactions, (c) apoptosis assays after *NNAT* re-expression and validation of *ALDH1A3* (d and e) methylation status and (f) *ALDH1A3* gene expression. **Supplemental document 1.** Description of the partition algorithm and the complete R script. Click here for file
