# Supplementary material for: Integrative DNA methylation and gene expression analysis in high-grade soft tissue sarcomas
Source: Genome Biol. 2013 Dec 17;14(12):r137. doi: 10.1186/gb-2013-14-12-r137 (PMC4054884; doi:10.1186/gb-2013-14-12-r137)
Supplement: Additional file 2: Figure S1 — Methylation-based regrouping of histopathological sarcoma subtypes. Figure S2. Workflow cross validation. Figure S3. Methylation status of the two CpG sites located on the X chromosome ((a) M-values, (b) binarized). (c) Importance of the CpG subgroups for classifying of the sarcoma subgroups. Figure S4. Heatmap of selected markers in the (a) primary collection and in (b)sarcoma cell lines. Figure S5. Bar plots of the DNA methylation of the minimal differential set between the different sarcoma clusters. Figure S6. Scatter plots of the minimal differential set. Figure S7. Comparison of (a) cluster 1 (DDLS), (b) cluster 3 (PLS) and (c) cluster 5 (MLS) versus fat. Figure S8. (a) Position of the CpG site and CpG islands in CDKN2A, (b and c) DNA methylation and gene expression profile of CDKN2A in the whole sarcoma collection. Figure S9. (a) Correlation between NNAT and UBC, (b) PubMed identifier (PMID) of the protein-wise interactions, (c) apoptosis assays after NNAT re-expression and validation of ALDH1A3 (d and e) methylation status and (f) ALDH1A3 gene expression. Supplemental document 1. Description of the partition algorithm and the complete R script. [file gb-2013-14-12-r137-S2.pdf]

Figure S1

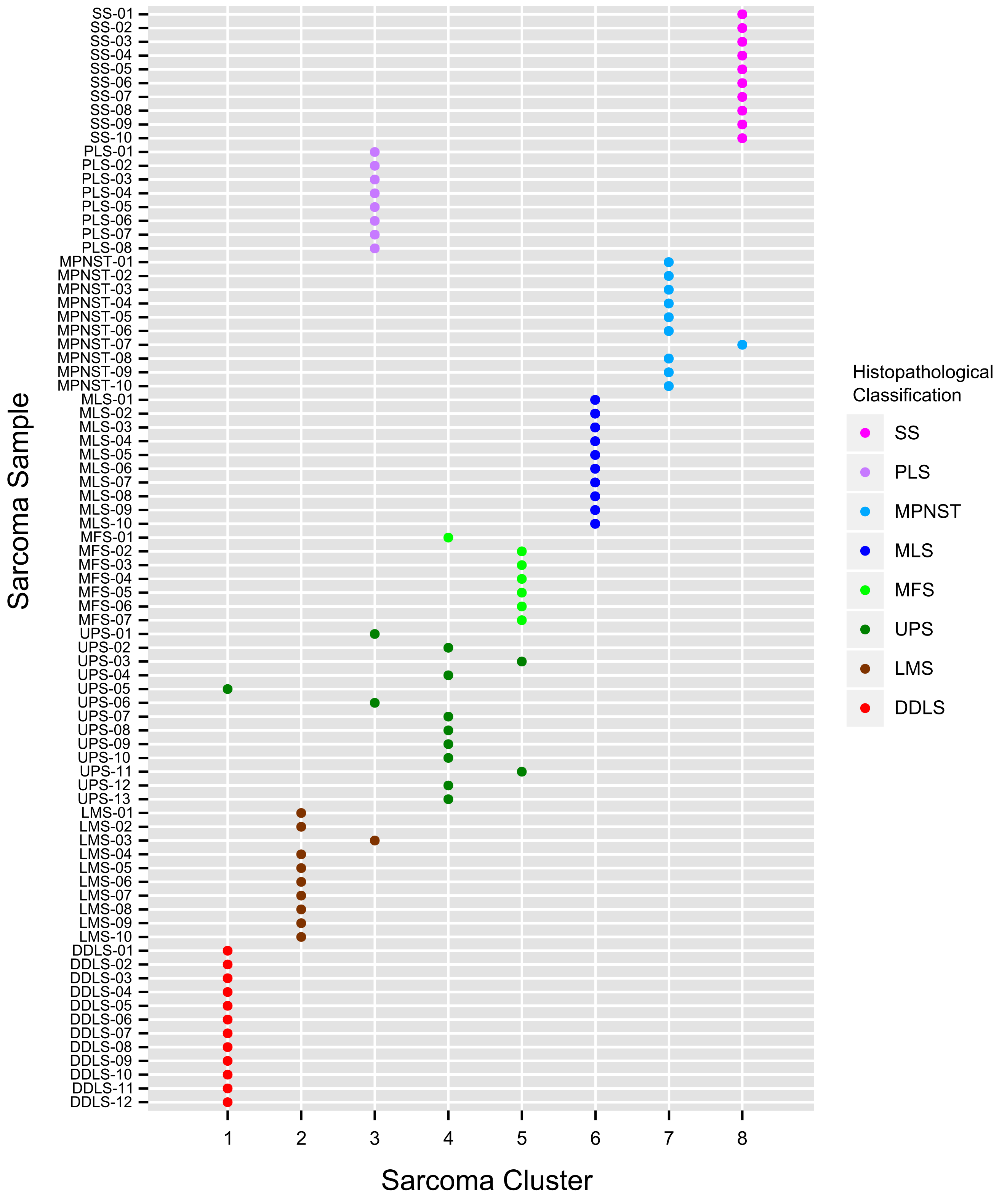

Sarcoma Sample
SS-01
SS-02
SS-03
SS-04
SS-05
SS-06
SS-07
SS-08
SS-09
SS-10
PLS-01
PLS-02
PLS-03
PLS-04
PLS-05
PLS-06
PLS-07
PLS-08
MPNST-01
MPNST-02
MPNST-03
MPNST-04
MPNST-05
MPNST-06
MPNST-07
MPNST-08
MPNST-09
MPNST-10
MLS-01
MLS-02
MLS-03
MLS-04
MLS-05
MLS-06
MLS-07
MLS-08
MLS-09
MLS-10
MFS-01
MFS-02
MFS-03
MFS-04
MFS-05
MFS-06
MFS-07
UPS-01
UPS-02
UPS-03
UPS-04
UPS-05
UPS-06
UPS-07
UPS-08
UPS-09
UPS-10
UPS-11
UPS-12
UPS-13
LMS-01
LMS-02
LMS-03
LMS-04
LMS-05
LMS-06
LMS-07
LMS-08
LMS-09
LMS-10
DDLS-01
DDLS-02
DDLS-03
DDLS-04
DDLS-05
DDLS-06
DDLS-07
DDLS-08
DDLS-09
DDLS-10
DDLS-11
DDLS-12
1
2
3
4
5
6
7
8
Sarcoma Cluster
Histopathological Classification
SS
PLS
MPNST
MLS
MFS
UPS
LMS
DDLS

Figure S2

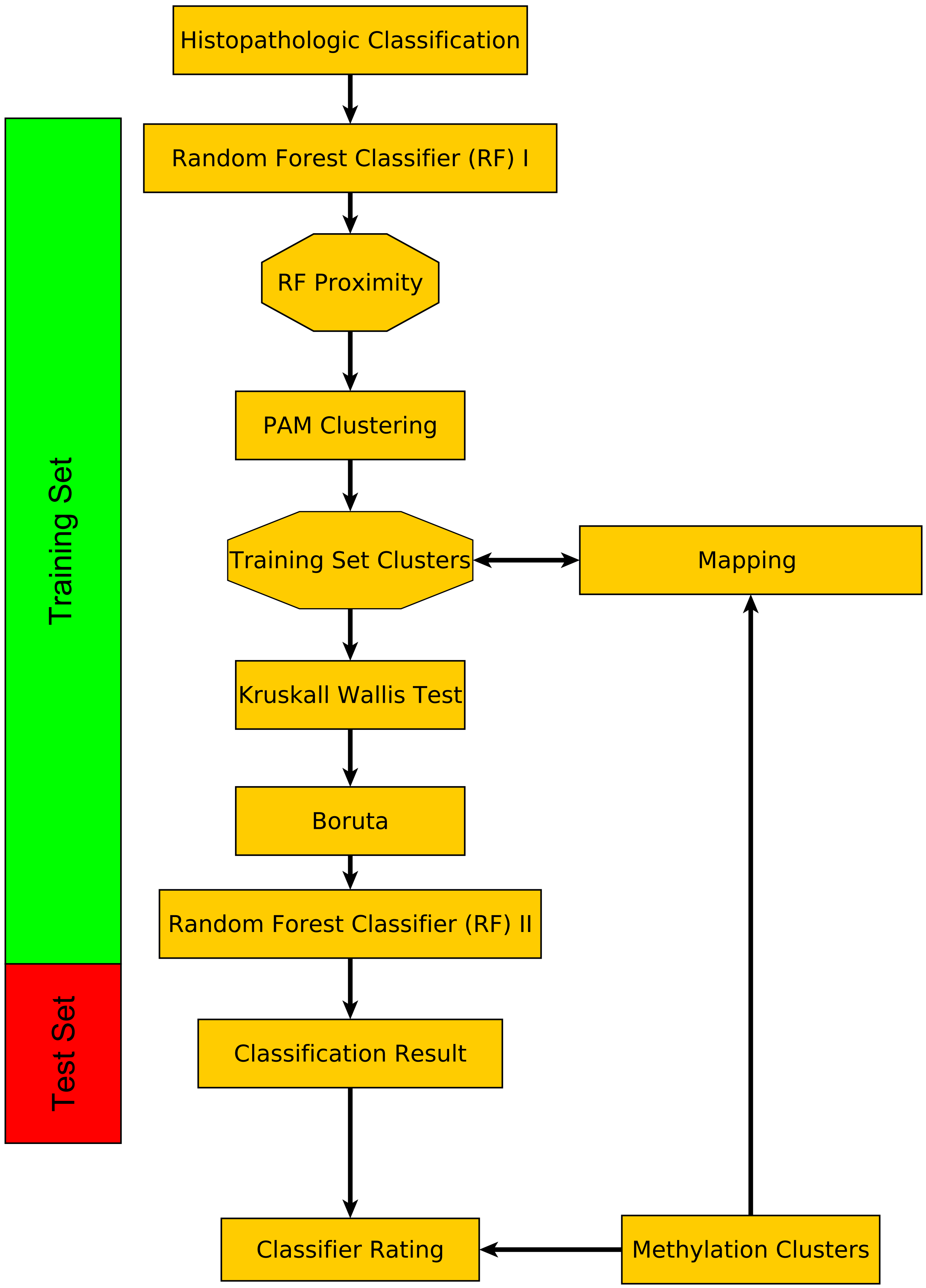
Histopathologic Classification
Random Forest Classifier (RF) I
RF Proximity
PAM Clustering
Training Set Clusters
Mapping
Kruskall Wallis Test
Boruta
Random Forest Classifier (RF) II
Classification Result
Classifier Rating
Methylation Clusters
Training Set
Test Set

Figure S3

(a)

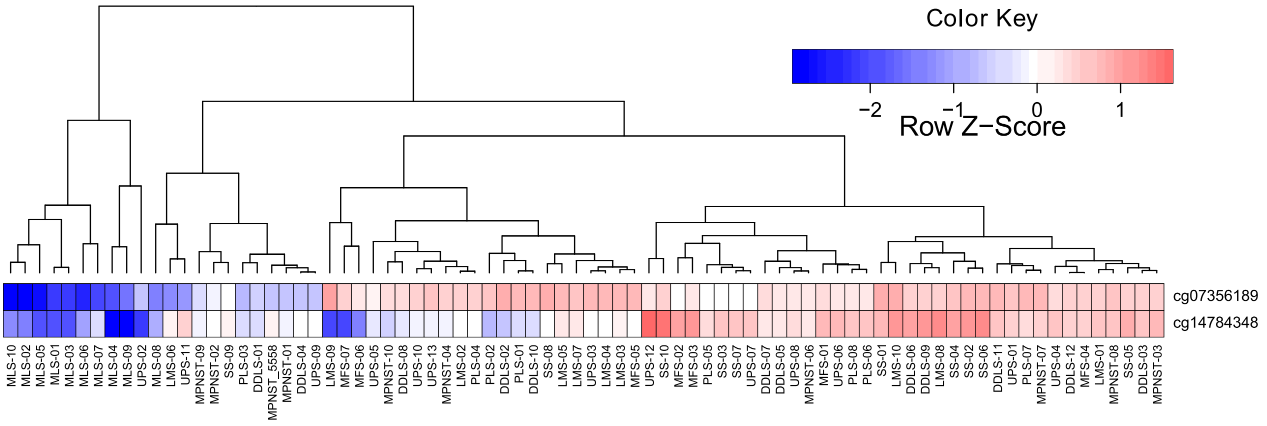

(b)

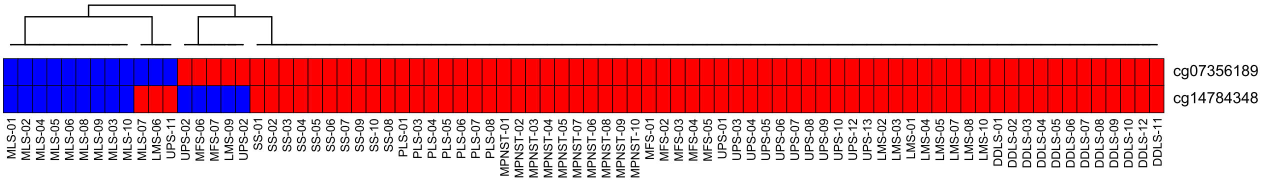

(c)

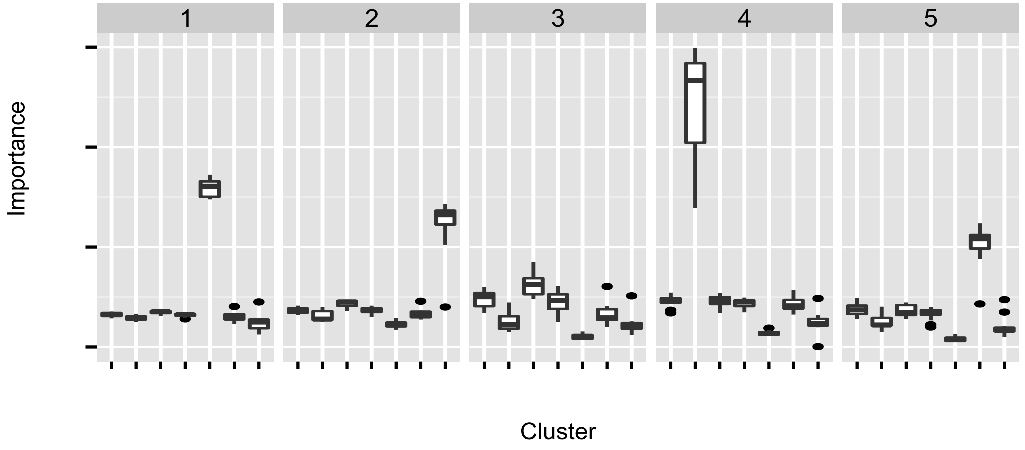

Figure S4

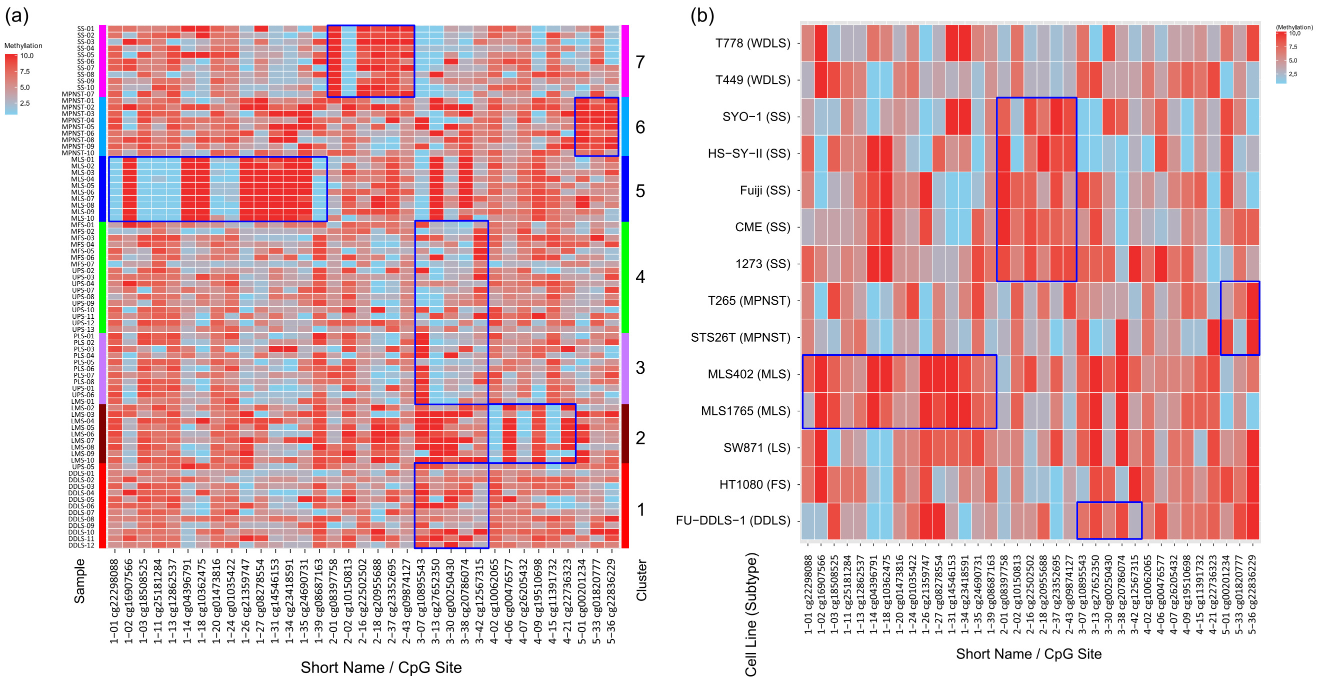
(a)
Methylation
10,0
7,5
5,0
2,5
Sample
Short Name / CpG Site
Cluster
7
6
5
4
3
2
1
(b)
T778 (WDLS)
T449 (WDLS)
SYO−1 (SS)
HS−SY−II (SS)
Fuiji (SS)
CME (SS)
1273 (SS)
T265 (MPNST)
STS26T (MPNST)
MLS402 (MLS)
MLS1765 (MLS)
SW871 (LS)
HT1080 (FS)
FU−DDLS−1 (DDLS)
Cell Line (Subtype)
Short Name / CpG Site

Figure S5

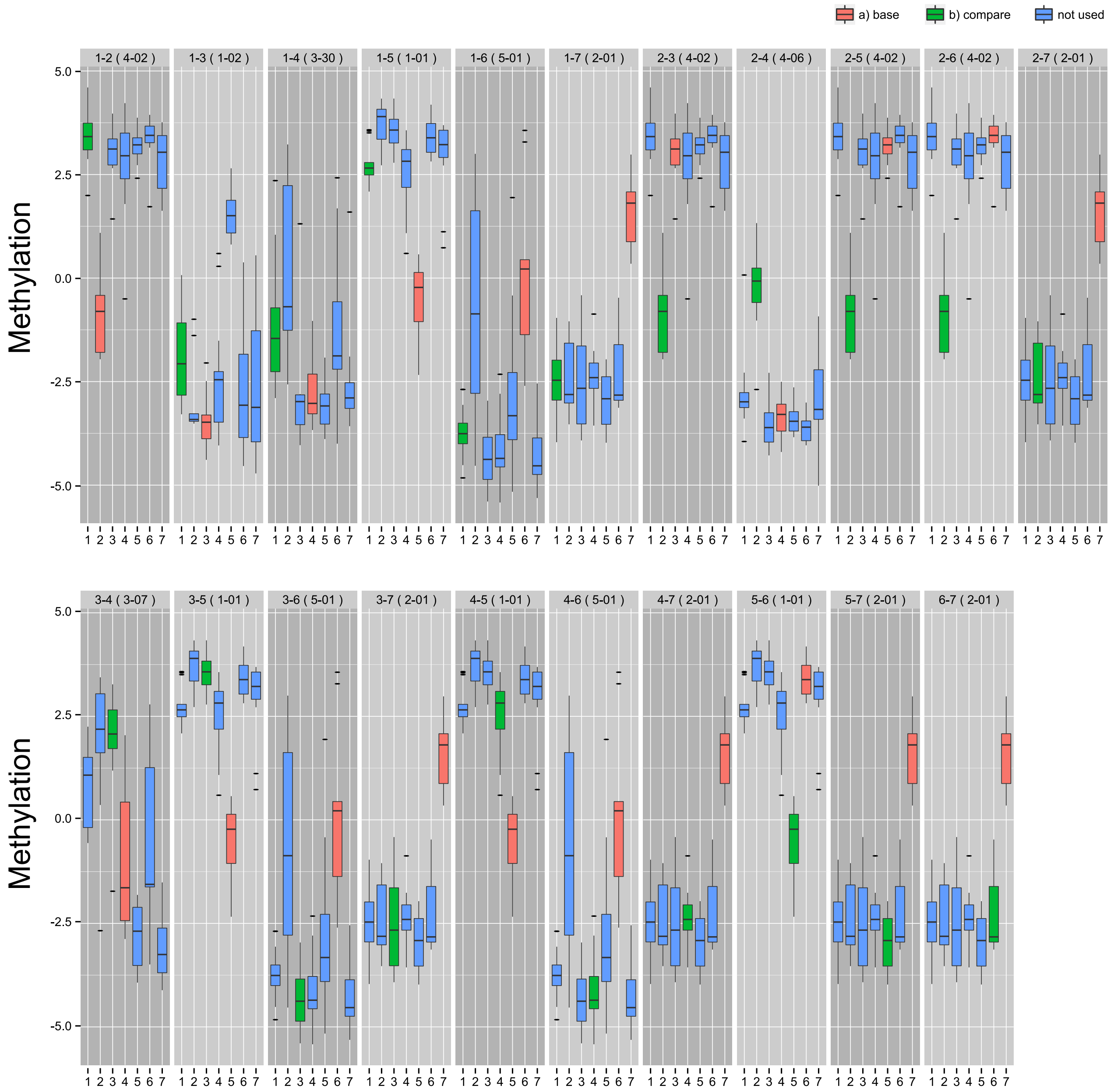

a) base
b) compare
not used
Methylation
1-2 (4-02)
1-3 (1-02)
1-4 (3-30)
1-5 (1-01)
1-6 (5-01)
1-7 (2-01)
2-3 (4-02)
2-4 (4-06)
2-5 (4-02)
2-6 (4-02)
2-7 (2-01)
3-4 (3-07)
3-5 (1-01)
3-6 (5-01)
3-7 (2-01)
4-5 (1-01)
4-6 (5-01)
4-7 (2-01)
5-6 (1-01)
5-7 (2-01)
6-7 (2-01)
5.0
2.5
0.0
-2.5
-5.0
1 2 3 4 5 6 7

Figure S6

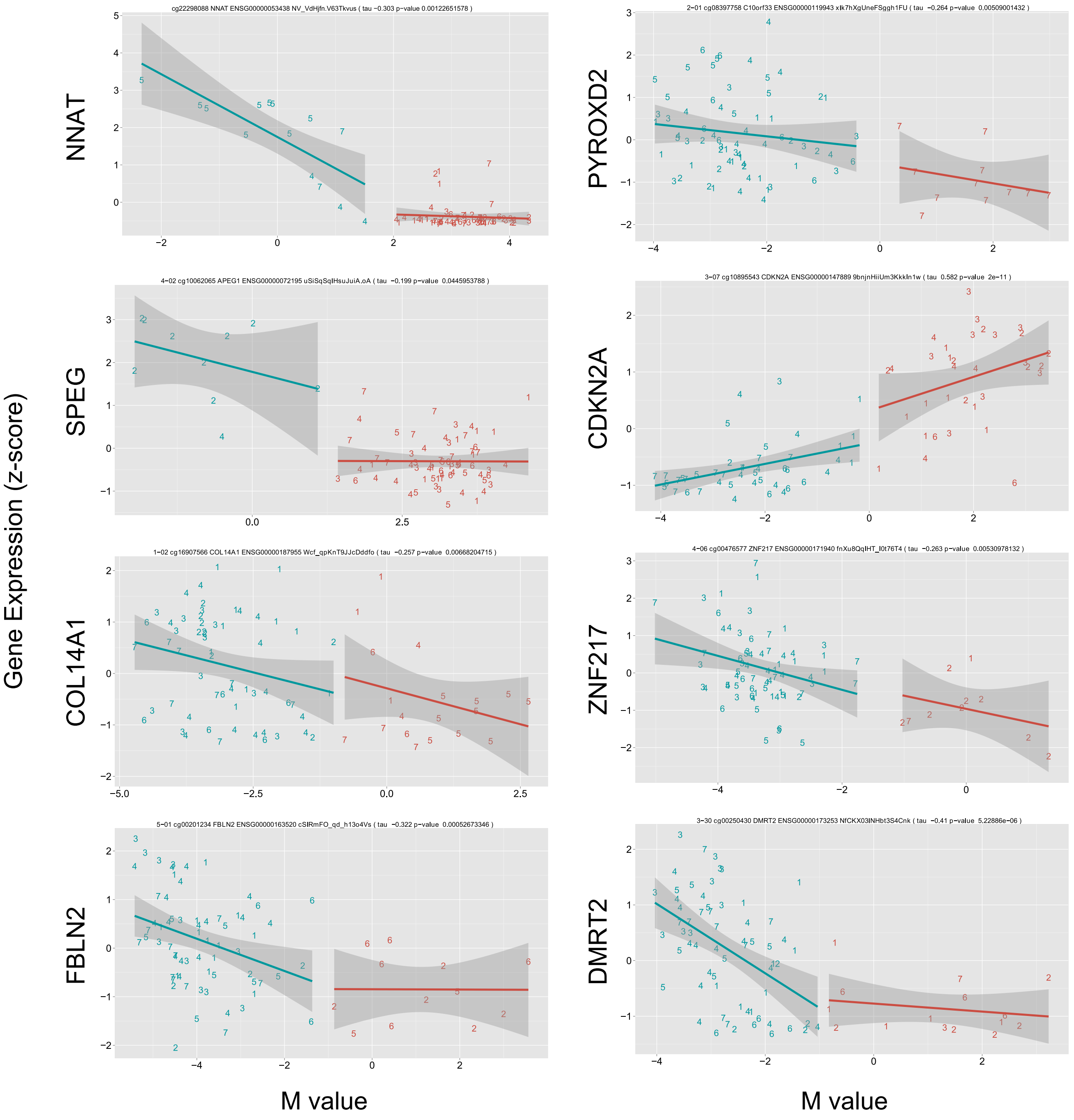

Gene Expression (z-score)
NNAT
cg22298088 NNAT ENSG00000053438 NV_VdHjfn.V63Tkvus (tau −0.303 p−value 0.00122651578)
PYROXD2
2−01 cg08397758 C10orf33 ENSG00000119943 xlk7hXgUneFSggh1FU (tau −0.264 p−value 0.00509001432)
SPEG
4−02 cg10062065 APEG1 ENSG00000072195 uSiSqSqIHsuJuiA.oA (tau −0.199 p−value 0.0445953788)
CDKN2A
3−07 cg10895543 CDKN2A ENSG00000147889 9bnjnHiiUm3KkkIn1w (tau 0.582 p−value 2e−11)
COL14A1
1−02 cg16907566 COL14A1 ENSG00000187955 Wcf_qpKnT9JJcDddfo (tau −0.257 p−value 0.00668204715)
ZNF217
4−06 cg00476577 ZNF217 ENSG00000171940 fnXu8QqIHT_I0t76T4 (tau −0.263 p−value 0.00530978132)
FBLN2
5−01 cg00201234 FBLN2 ENSG00000163520 cSIRmFO_qd_h13o4Vs (tau −0.322 p−value 0.00052673346)
DMRT2
3−30 cg00250430 DMRT2 ENSG00000173253 NfCKX03INHbt3S4Cnk (tau −0.41 p−value 5.22886e−06)
M value
M value

Figure S7

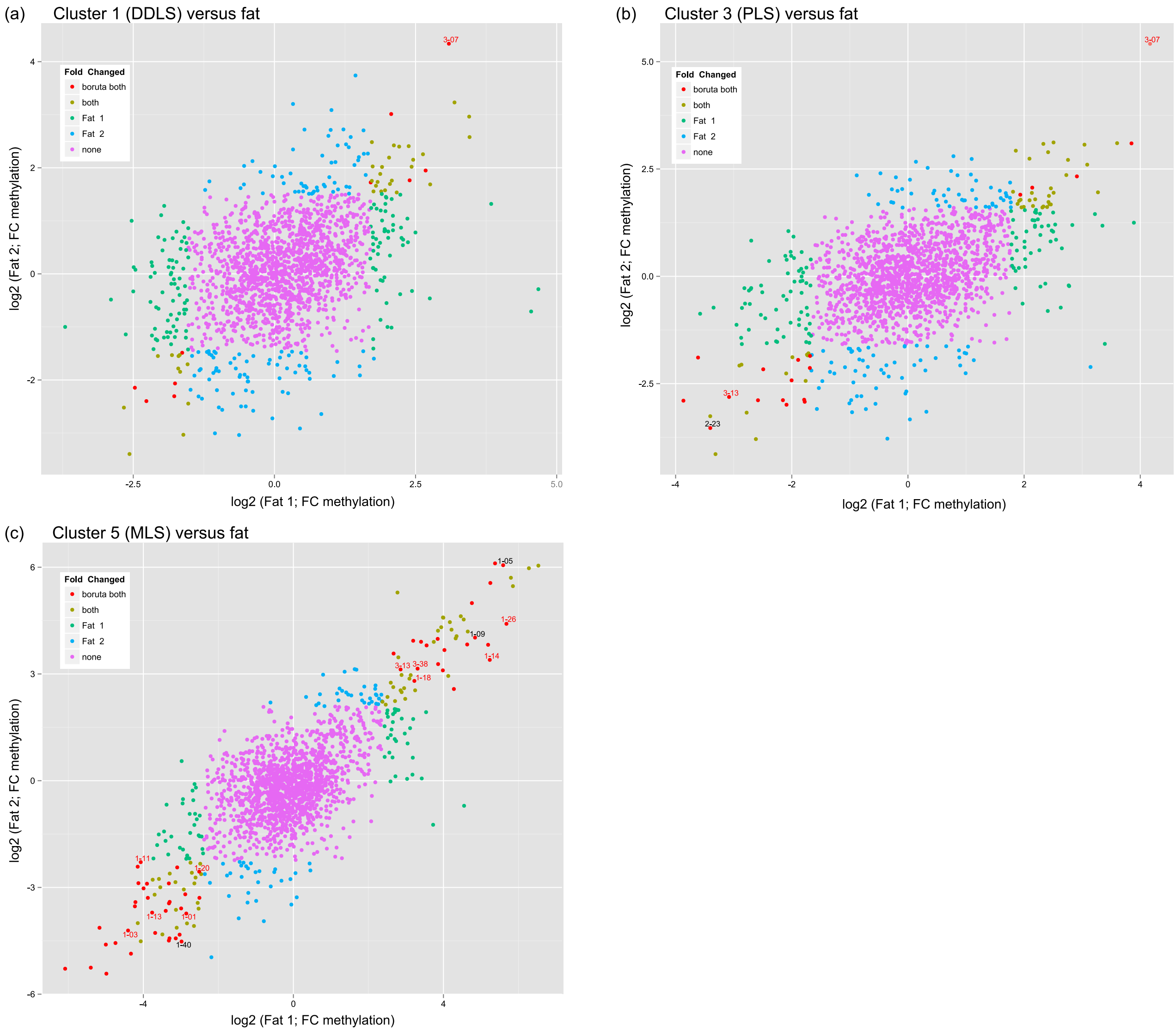
(a)
Cluster 1 (DDLS) versus fat
Fold Changed
boruta both
both
Fat 1
Fat 2
none
3-07
log2 (Fat 2; FC methylation)
log2 (Fat 1; FC methylation)
(b)
Cluster 3 (PLS) versus fat
Fold Changed
boruta both
both
Fat 1
Fat 2
none
3-07
3-13
2-23
log2 (Fat 2; FC methylation)
log2 (Fat 1; FC methylation)
(c)
Cluster 5 (MLS) versus fat
Fold Changed
boruta both
both
Fat 1
Fat 2
none
1-05
1-26
1-09
1-14
3-13
3-38
1-18
1-11
1-20
1-13
1-01
1-03
1-40
log2 (Fat 2; FC methylation)
log2 (Fat 1; FC methylation)

Figure S8

(a)

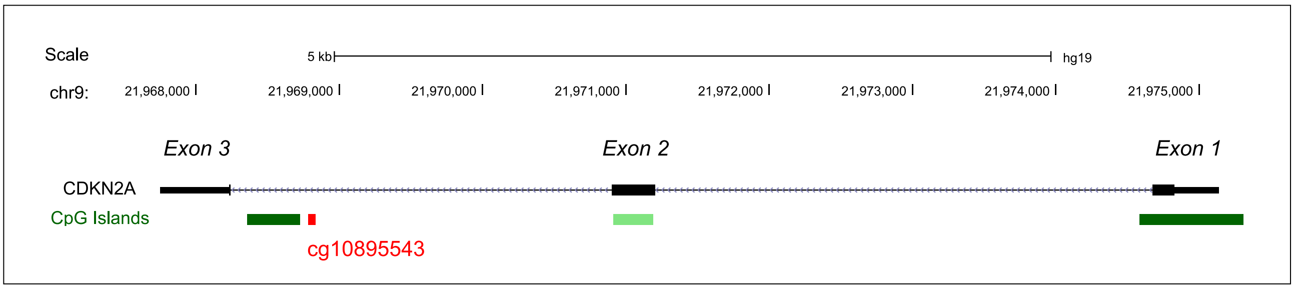

(b)

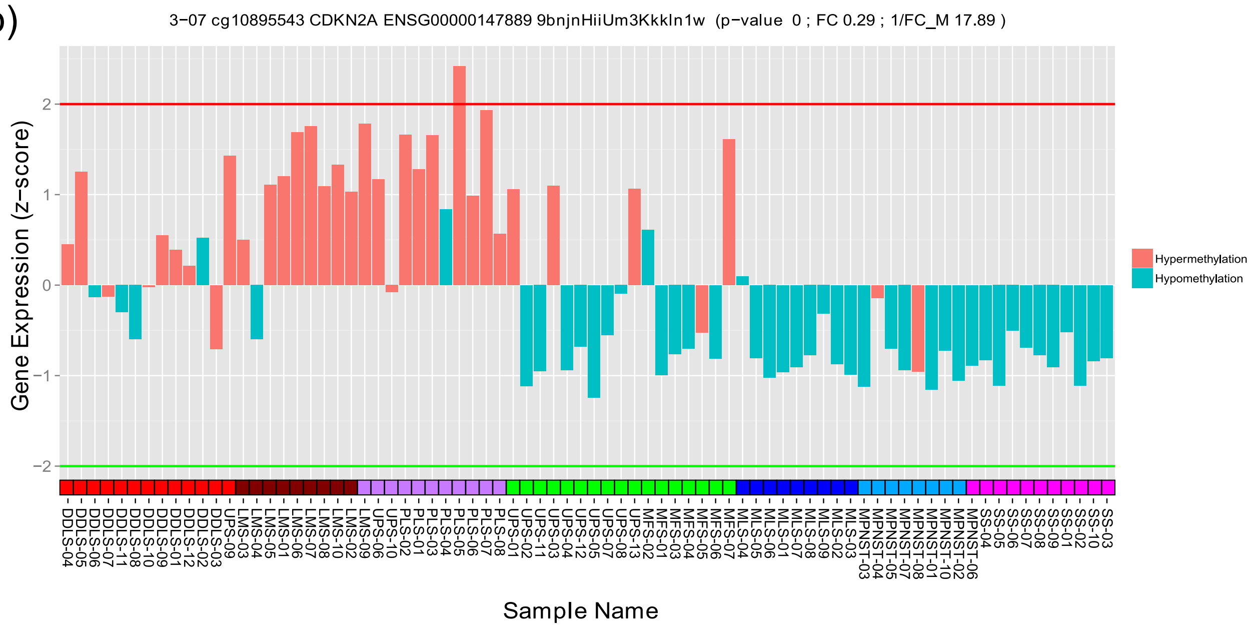

(c)

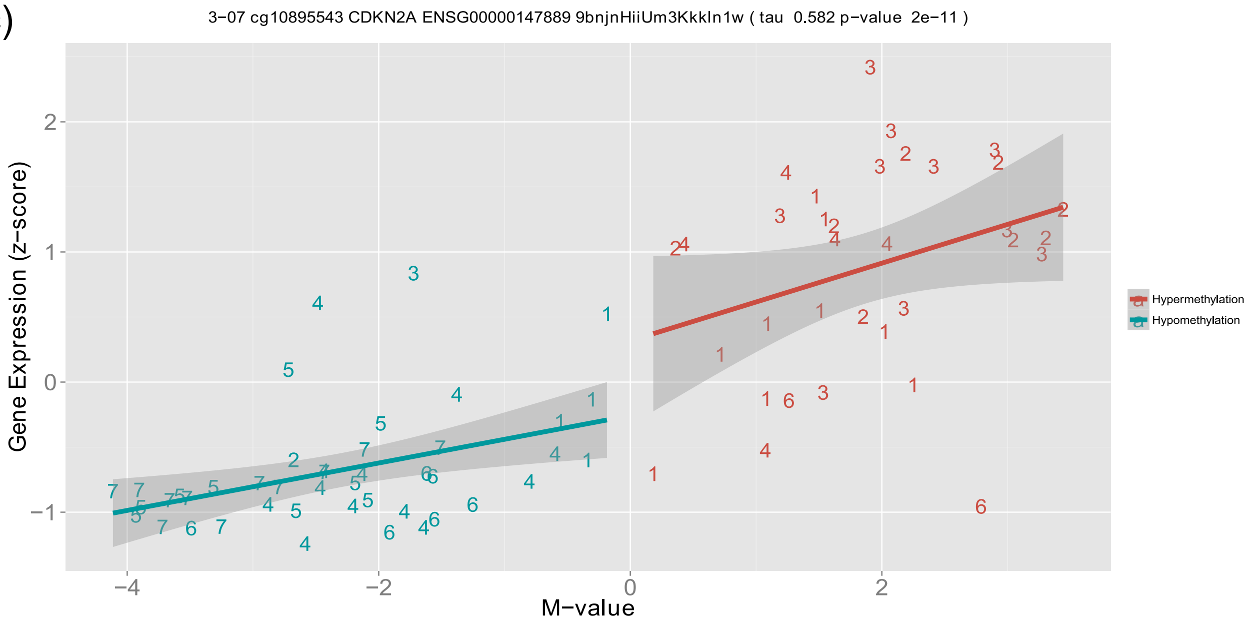

Figure S9

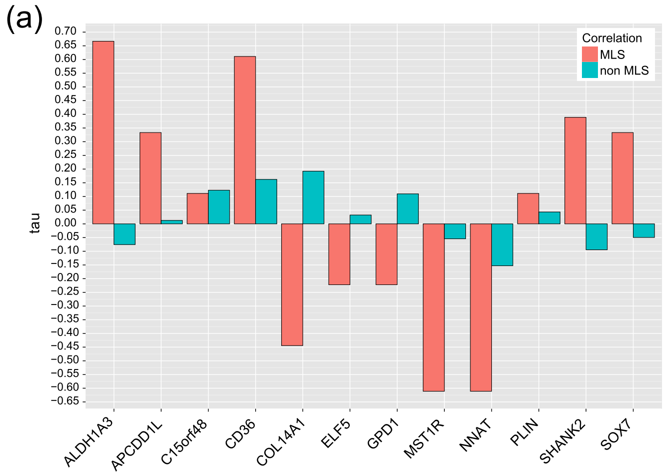

(b)

Gene Interaction	PMID				
NNAT - UBC	21742036				
PIK3R1 - UBC	11087752	18374639	2119048		
MST1R - UBC	12802274	16740632	21906983		
SRC - UBC	17936276	20584982	21139048	21911421	22447928
PLCG1 - UBC	17372230	18671761	21139048	21890473	21906983
ALDH1A3 -UBC	21906983				
YES1 - UBC	21139048	21890473			
COL14A1 - UBC	21139048				
CD36 - SRC	7521304				
PLCG1 - SRC	1689310	7510703	7682059	8657103	8885868
MST1R - SRC	16103119	16166096	8918464		
PIK3R1 - SRC	10487518	12210743	19861161	19903481	
SHANK2 - SRC	17474147				
PK3R1 - MST1R	8918464				
YES1 - MST1R	8918464				
PLCG1 - MST1R	8918464				
PIK3R1 - MST1R	8918464				
CD36 - YES1	1715582	7521304			
PLCG1 - SHANK2	17474147				
PIK3R1 - SHANK2	17474147				

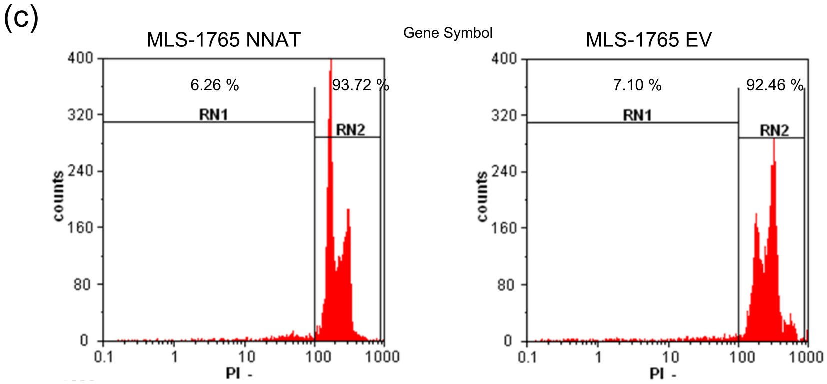

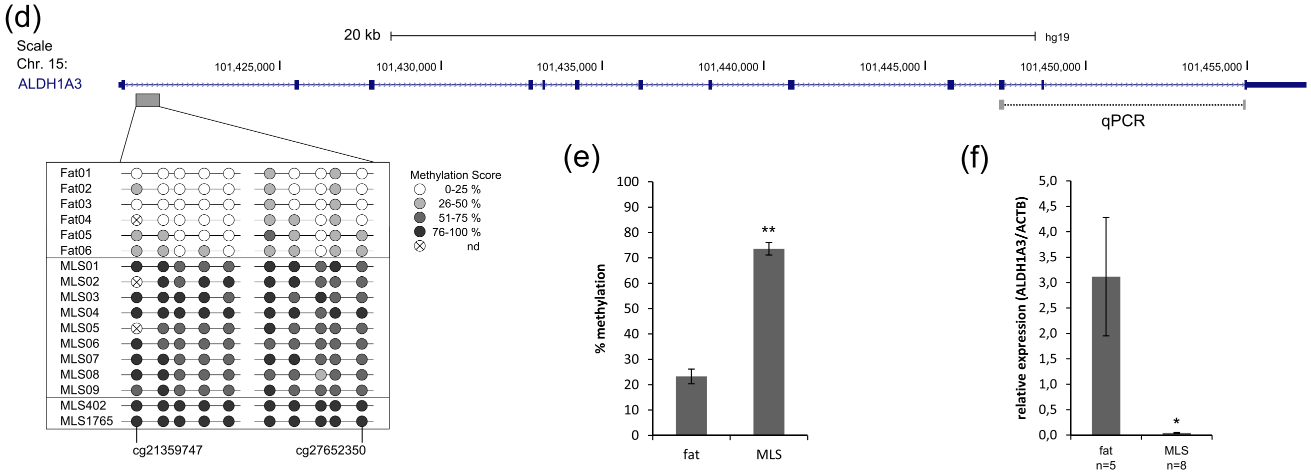

Supplemental Document 1

Analysis of methylation statuses

In general, two different types of probe-specific M-value profiles across the sample population can be expected:

1. Probes displaying a bimodal M-value profile, i. e. two sample subgroups, one with methylated and one with unmethylated DNA status at that locus.

2. Probes with a unimodal M-value profile, i. e. only DNA with either methylated or unmethylated status at that locus for the majority of the samples.

An algorithm to partition expression data (Bessarabova et al. 2010) was modified to determine whether a uni- or bimodal M-value distribution was present at any particular locus, and in the latter case, which M-value could be used to set as a threshold dividing the samples into the two subgroups. In brief, the M-values of all samples at a particular locus *x* (variable names in *italics* correspond to variables in the R script provided below) were sorted by value. Iteratively, each M-value of the sorted list sort was chosen as a threshold and the average (*lower, upper*) and sum of square deviations (*sum squares*) of the resulting subgroups were calculated. The M-value leading to the lowest sum of square deviations was chosen as the threshold value at that particular locus. In order to increase the robustness of this partition procedure to outliers, only threshold values leading to a group size of more than 5% of the entire sample population were accepted (*threshold.outliers*). In addition, a value *tau* was determined for each locus, serving as a marker for the level of bimodality. Only if *tau* exceeded a predefined threshold *tau.threshold*, the sample distribution was considered bimodal.

In case of bimodal M-value distribution, the samples were partitioned into two groups according to the calculated *threshold.outliers*. In order to ensure a distinct and reliable separation of the two subgroups, samples with M-values in an interval of 1/8 of the M-value

range around the *threshold.outliers* were excluded in the next analysis step. Probes where those preselected samples accounted for at least 80% of the entire sample population were extracted and all samples' methylation values were binarized according to the *threshold.outliers* value: 0 for unmethylated (less than threshold) and 1 for methylated (greater than threshold).

Partition Algorithm (R script)

```
myPartition = function(x,tau.threshold) {
            sort = sort(x, na.last=NA)
   sum_squares = vector(mode = "numeric", length = (length(sort) - 1))
   lower = vector(mode = "numeric", length = (length(sort) - 1))
   upper = vector(mode = "numeric", length = (length(sort) - 1))
   for (k in 1:length(sort)) {
        lower[k] = sum(sort[1:k])/k
        upper[k] = sum(sort[(k+1):length(sort)])/(length(sort)-k)
        sum_squares[k] = sum((sort[1:k]-lower[k])^2) + sum((sort[(k+1):length(sort)]-
upper[k])^2)
     }
   threshold_position = as.integer(which(sum_squares == min(sum_squares, na.rm =
TRUE)))
   threshold = (upper[threshold_position] + lower[threshold_position])/2
   if ((length(x[x<threshold])/length(x) || length(x[x>threshold])/length(x))<0.05) {
                 while ((length(x[x<threshold])/length(x) ||
length(x[x>threshold])/length(x))<0.05) {
                        sum_squares_outliers = sum_squares[-which(sum_squares_outliers ==
min(sum_squares_outliers, na.rm = TRUE))]
                        threshold_position_outliers = as.integer(which(sum_squares_outliers ==
min(sum_squares_outliers, na.rm = TRUE)))
                        threshold_outliers = (upper[threshold_position_outliers] +
lower[threshold_position_outliers]) / 2
            }
   }else{
```

```
                threshold_outliers = threshold
                threshold_position_outliers = threshold_position
    }
   tau = (upper[threshold_position_outliers] - lower[threshold_position_outliers]) /
sqrt((threshold_outliers)^2 / length(sort))
if (tau > tau.threshold) {
        if (range(x)[1] < 0) {
                keep = ((x < (threshold_outliers - ((range(x)[2] + abs(range(x)[1])) / 8))) | (x >
(threshold_outliers + (range(x)[2] + abs(range(x)[1])) / 8)))
        }else{
                keep = ((x < (threshold_outliers - ((range(x)[2] - range(x)[1]) / 8))) | (x >
(threshold_outliers + ((range(x)[2] - range(x)[1]) / 8))))
        }
   }
if (tau < tau.threshold) {
                keep = rep(FALSE, length(x))
        }
return(c(sum_squares[threshold_position_outliers], threshold_outliers, tau, keep))
}
```

References

Bessarabova M, Kirillov E, Shi W, Bugrim A, Nikolsky Y, Nikolskaya T. 2010. Bimodal gene expression patterns in breast cancer. *BMC Genomics* 11: S8.
